# Supplementary material for: Novel AAV capsids for intravitreal gene therapy of photoreceptor disorders
Source: EMBO Mol Med. 2021 Feb 22;13(4):e13392. doi: 10.15252/emmm.202013392 (PMC8033523; doi:10.15252/emmm.202013392)
Supplement: Supplementary file 1 — Appendix [file EMMM-13-e13392-s010.pdf]

Appendix

Content: Appendix Table S1

Figure 1 panel F: Ordinary one-way ANOVA (summary P value 0.0044)
AAV2 n=4 , 7m8 n=3 , GL n=5 , NN n=3

Holm-Šídák's multiple comparisons test	Mean Diff.	Below threshold?	Summary	Adjusted P Value
AAV2 vs. GL	-12.32	Yes	**	0.0030
AAV2 vs. NN	-11.01	Yes	*	0.0102
AAV2 vs. 7m8	-4.453	No	ns	0.1862

Figure 2 panel B: Ordinary one-way ANOVA (summary P value 0.0002)
AAV2 n=4, 7m8 n=4 , GL n=3 , NN n=4

Holm-Šídák's multiple comparisons test	Mean Diff.	Below threshold?	Summary	Adjusted P Value
AAV2 vs. 7m8	-4.020	No	ns	0.2725
AAV2 vs. GL	-8.072	Yes	*	0.0427
AAV2 vs. NN	-17.08	Yes	***	0.0002
7m8 vs. GL	-4.052	No	ns	0.2725
7m8 vs. NN	-13.06	Yes	**	0.0018
GL vs. NN	-9.009	Yes	*	0.0313

Figure 6 panel C: Multiple unpaired t-tests using Holm-Šídák method comparing IVT/GL Vs UN
IVT/GL n=9, UN n=8, WT n=6

Condition	Below threshold?	P value	Mean of IVT / GL	Mean of UN
1 cd.s/m²	Yes	0.002926	9.941	1.617
3 cd.s/m²	Yes	0.000009	12.60	-0.6551
10 cd.s/m²	Yes	<0.000001	14.85	-0.8085

Multiple unpaired t-tests using Holm-Šídák method comparing IVT/GL Vs WT

Condition	Below threshold?	P value	Mean of IVT / GL	Mean of WT
1 cd.s/m²	Yes	<0.000001	9.941	54.42
3 cd.s/m²	Yes	<0.000001	12.60	87.77
10 cd.s/m²	Yes	<0.000001	14.85	118.1

Expanded View Figure 3 panel F: Ordinary one-way ANOVA (summary P value 0.0037)
AAV2 n=3, AAV2.GL n=3, AAV2.NN n=3

Holm-Šídák's multiple comparisons test	Mean Diff.	Below threshold?	Summary	Adjusted P Value
AAV2 vs. AAV2.GL	-14.93	Yes	**	0.0025
AAV2 vs. AAV2.NN	-8.629	Yes	*	0.0164

Appendix Table S1: Statistical Analysis Details
