# Supplementary material for: Novel AAV capsids for intravitreal gene therapy of photoreceptor disorders
Source: EMBO Mol Med. 2021 Feb 22;13(4):e13392. doi: 10.15252/emmm.202013392 (PMC8033523; doi:10.15252/emmm.202013392)
Supplement: Supplementary file 2 — Expanded View Figures PDF [file EMMM-13-e13392-s009.pdf]

Expanded View Figures

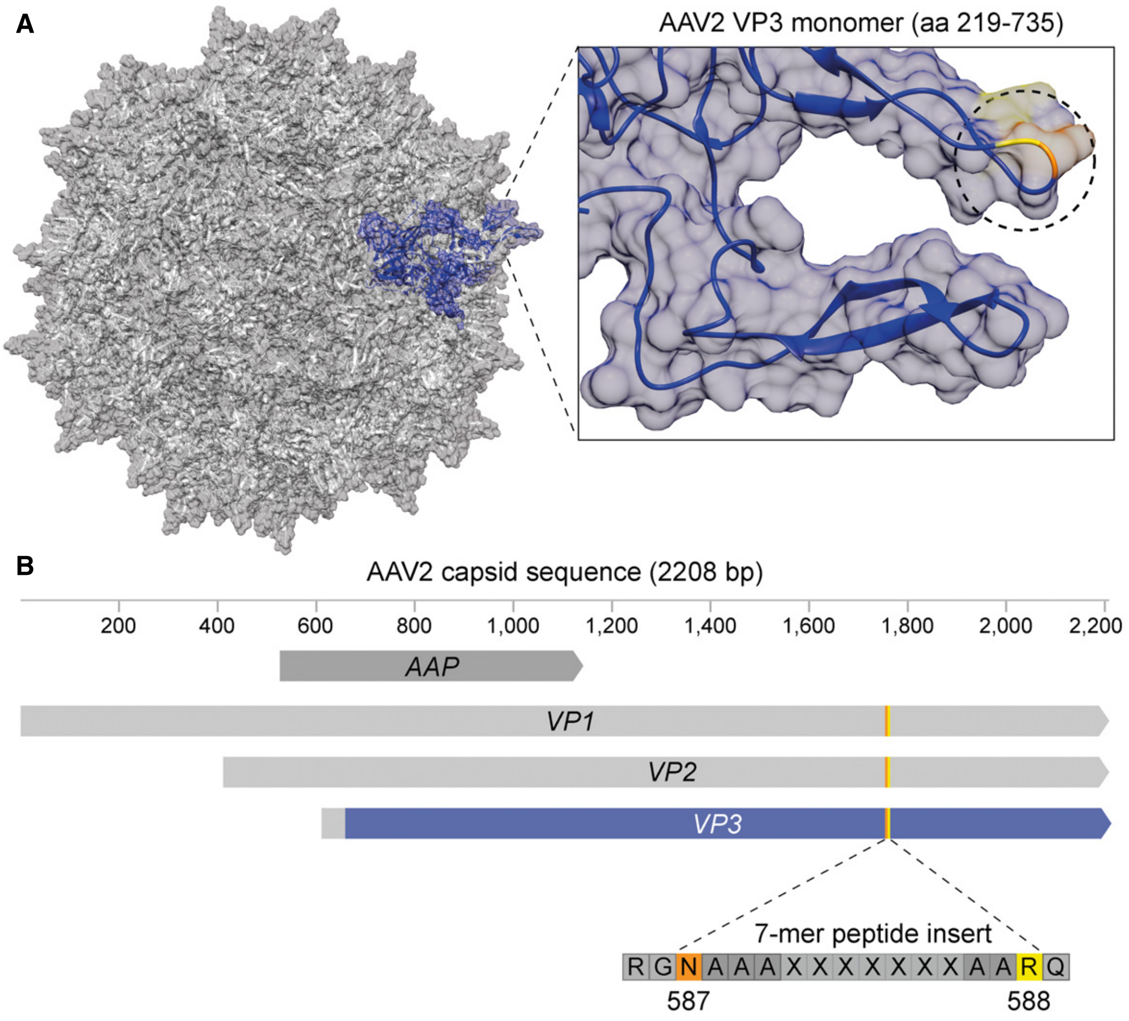

Figure EV1. Schematic summary of peptide-display capsid diversification strategy.

A Depiction of the parental AAV2 capsid structure (PDB 6IH9) with a single VP3 monomer (amino acids (aa) 219–735) coloured blue. On the right, the blue monomer is shown in focus and the unmodified insertion position I-587 is circled with the residues N587 and R588 highlighted in orange and yellow, respectively.

B Schematic of AAV2 capsid sequence with the constituent genes, i.e. assembly activating protein (AAP) and viral protein (VP) 1–3. The part of VP3-coloured blue represents the sequence of the structural monomer depicted in (A) with the same colour. The orange/yellow line in VP3 and schematic below indicate the site of insertion of the random 7-mer peptides between N587 (orange) and R588 (yellow) flanked by alanine linkers.

 © 2021 The Authors

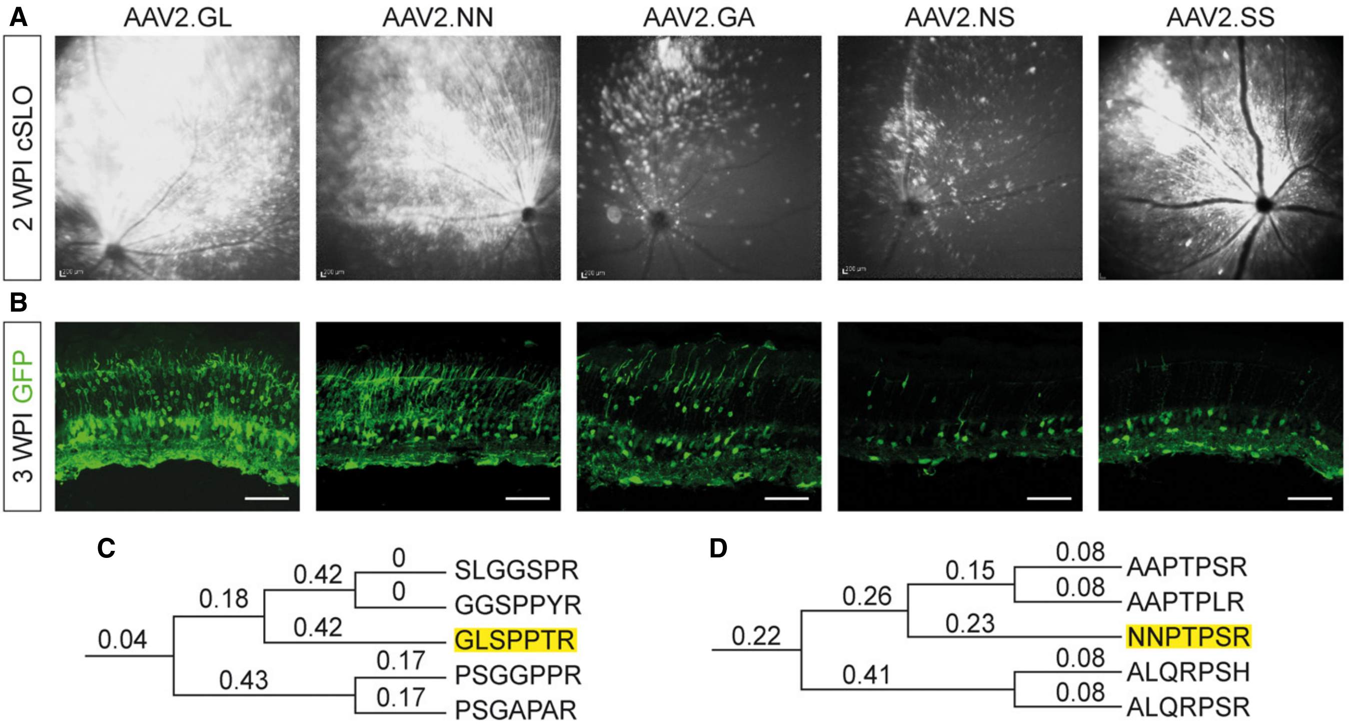

Figure EV2. Selection of optimal candidate from vectorised rAAV variants.

Comparative study between vectorised novel capsid variants AAV2.GL, AAV2.NN, AAV2.GA, AAV2.NS and AAV2.SS packaged with sc-CMV-eGFP; intravitreal administration of 2E9 vg.

A cSLO reporting transduction and spread of vectors in the murine retina after 2 WPI.

B Confocal scans of native eGFP fluorescence after 3WPI. Scale bar: 50 µm. Acquisition settings were kept constant for all samples. GFP = eGFP.

C, D Phylogenetic tree of a selection of AAV variants detected via NGS in the cone (C) and rod (D) DNA from screening round 3. The amino acid sequences of peptide inserts were aligned, and a phylogenetic tree was constructed using the maximum likelihood method. Numbers indicate bootstrap values, and the peptides corresponding to AAV2.GL and AAV2.NN are highlighted.

© 2021 The Authors

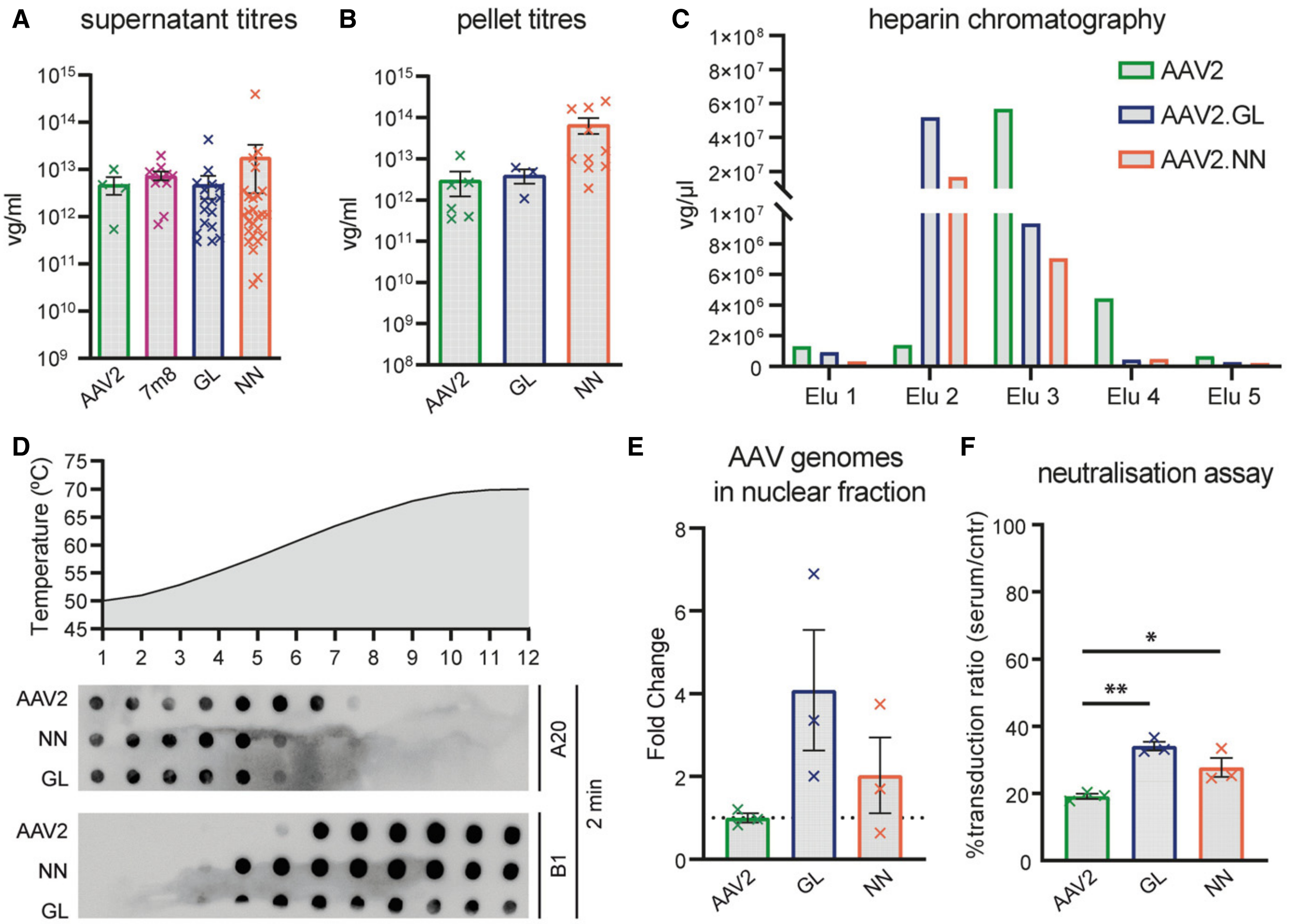

Figure EV3. ***In vitro* characterisation of AAV2.GL and AAV2.NN.**

A Production yield of capsids AAV2.GL, AAV2.NN, AAV2 and AAV2.7m8 quantified by qPCR as viral genomes (vg)/ml, harvested from HEK293T supernatant during standard AAV production. Biological replicates AAV2 $n = 4$, AAV2.7m8 $n = 11$, AAV2.GL $n = 17$, AAV2.NN $n = 26$. Bars show mean ± SEM.

B Same as for (A) with comparison only to AAV2, harvested from HEK293T cell pellet. Biological replicates AAV2 $n = 6$, AAV2.GL $n = 3$, AAV2.NN $n = 10$. Bars show mean ± SEM.

C Heparin affinity assay performed using chromatography. Consecutive eluates from the heparin column were used to detect the presence of AAV2, AAV2.GL and AAV2.NN, indicating binding strength.

D Capsid stability assay comparing AAV2 to AAV2.GL and AAV2.NN. Dot plots of AAV particles stained for A20 and B1, following incubation for 2 min, at consecutive degeneration steps in the form of increasing temperature.

E qPCR quantification of rAAV genomes in the nuclear fraction of transduced HeLa cells, normalised to cellular gDNA. Biological replicates $n = 3$. Bars show mean ± SEM.

F Neutralisation assay using HeLa cells after pre-incubating AAV2, AAV2.GL and AAV2.NN with human serum. rAAV vector-induced eGFP signal served as transduction reporter. The % ratio of transduced cells after pre-incubating AAVs with serum relative to control (cntr) unchallenged AAVs is shown. Biological replicates $n = 3$. Bars show mean ± SEM. One-way ANOVA with Holm–Sidak multiple comparisons test was performed. $*P \leq 0.05$, $**P \leq 0.01$. Detailed statistical analysis in Appendix Table S1.

Source data are available online for this figure.

© 2021 The Authors

Figure EV4. Structural modelling of novel capsids.

Comparative modelling of novel capsid variants (Robetta.bakerlab.org/submit) based on AAV2 (PDB 6IH9) processed using Chimera (Pettersen *et al*, 2004).

A, B Full capsid of AAV2.GL and AAV2.NN, respectively, with the 7-mer peptide insert in red and the alanine linker in yellow.

C, D Overlapped structural monomers of AAV2.GL and AAV2.NN, respectively, shown in blue, overlapped to AAV2, shown in white. 7-mer peptide insert is shown in red and the alanine linker in yellow. Key residues N587 and R588 shown in green.

E, F Focused view on the variable loops 3 and 4 of the comparative overlaps in (C) and (D), respectively. The colour scheme is consistent as before.

G, H Focused view on the variable loops 3 and 4 of the comparative overlap of structural monomers AAV2.GL and AAV2.NN, respectively, with AAV2.7m8 (PDB 6U0R) (Bennett *et al*, 2020). The colour scheme for AAV2.GL (G) and AAV2.NN (H) is as before. The AAV2.7m8 structural monomer is shown in black, and the peptide displayed on loop 4 is shown in cyan.

Source data are available online for this figure.

© 2021 The Authors

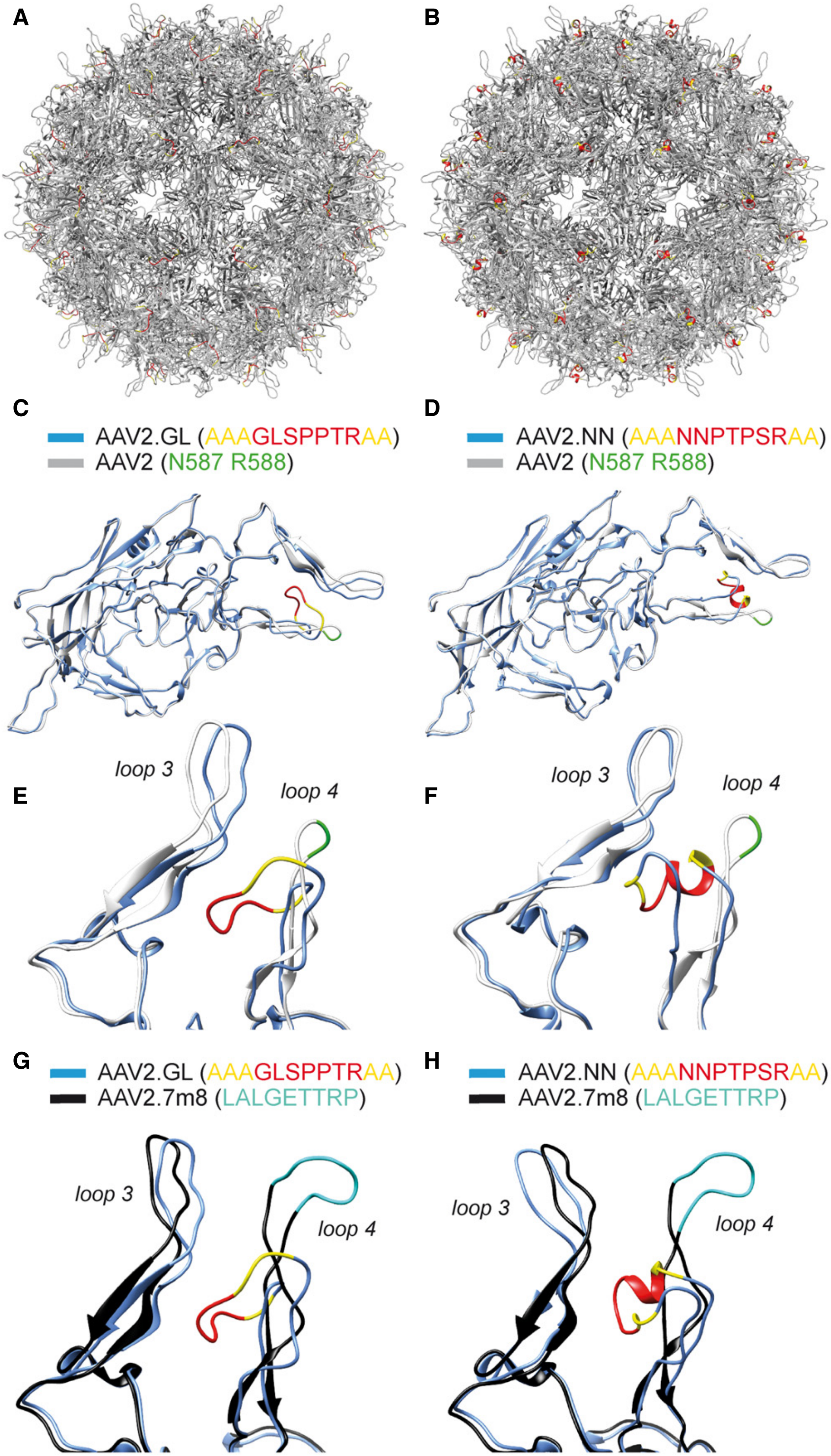

Figure EV4.

© 2021 The Authors

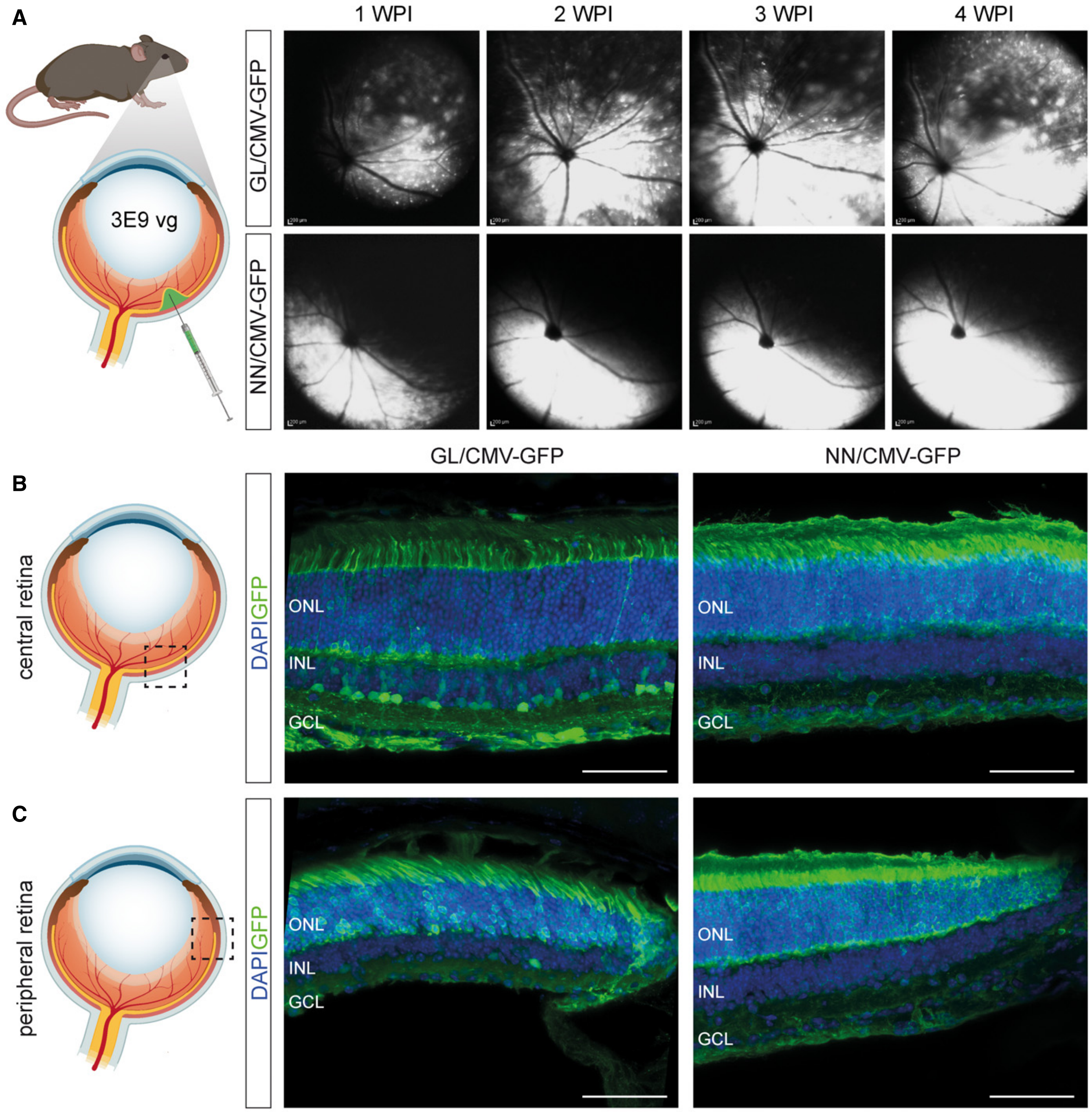

Figure EV5. Subretinal injection of AAV2.GL and AAV2.NN in mouse.

Vector mediated eGFP expression and lateral spreading after subretinal injection in 1-month-old mouse retinas.

A Schematic depiction of subretinal injection in wild-type mice followed by weekly in vivo cSLO examinations of the mouse retina from 1 to 4 weeks post-injection (WPI) of 3E9 total vg (in 1 μl) of sc-CMV-eGFP packaged with AAV2.GL and AAV2.NN capsid variants.

B Schematic depiction of central locus shown in adjacent confocal scans of retinal cross sections immunolabelled for eGFP and DAPI, reporting transduction efficacy of AAV2.GL (left) and AAV2.NN (right).

C Schematic depiction of peripheral locus shown in adjacent immunolabelled confocal scans, as in (B).

Data information: Acquisition settings were kept constant for all samples. GFP = eGFP; ONL, outer nuclear layer; INL, inner nuclear layer; GCL, ganglion cell layer. Scale bar: 50 μm.
Source data are available online for this figure.

© 2021 The Authors
