# Supplementary material for: Novel AAV capsids for intravitreal gene therapy of photoreceptor disorders
Source: EMBO Mol Med. 2021 Feb 22;13(4):e13392. doi: 10.15252/emmm.202013392 (PMC8033523; doi:10.15252/emmm.202013392)
Supplement: Supplementary file 3 — Source Data for Expanded View [file EMMM-13-e13392-s008.zip › emmm202013392-sup-0009-SDataEVFig3/Source_Data_File_for_EV_Fig_3/Expanded_View_Figure_3_Dot_Plot.pptx]

## Slide 1
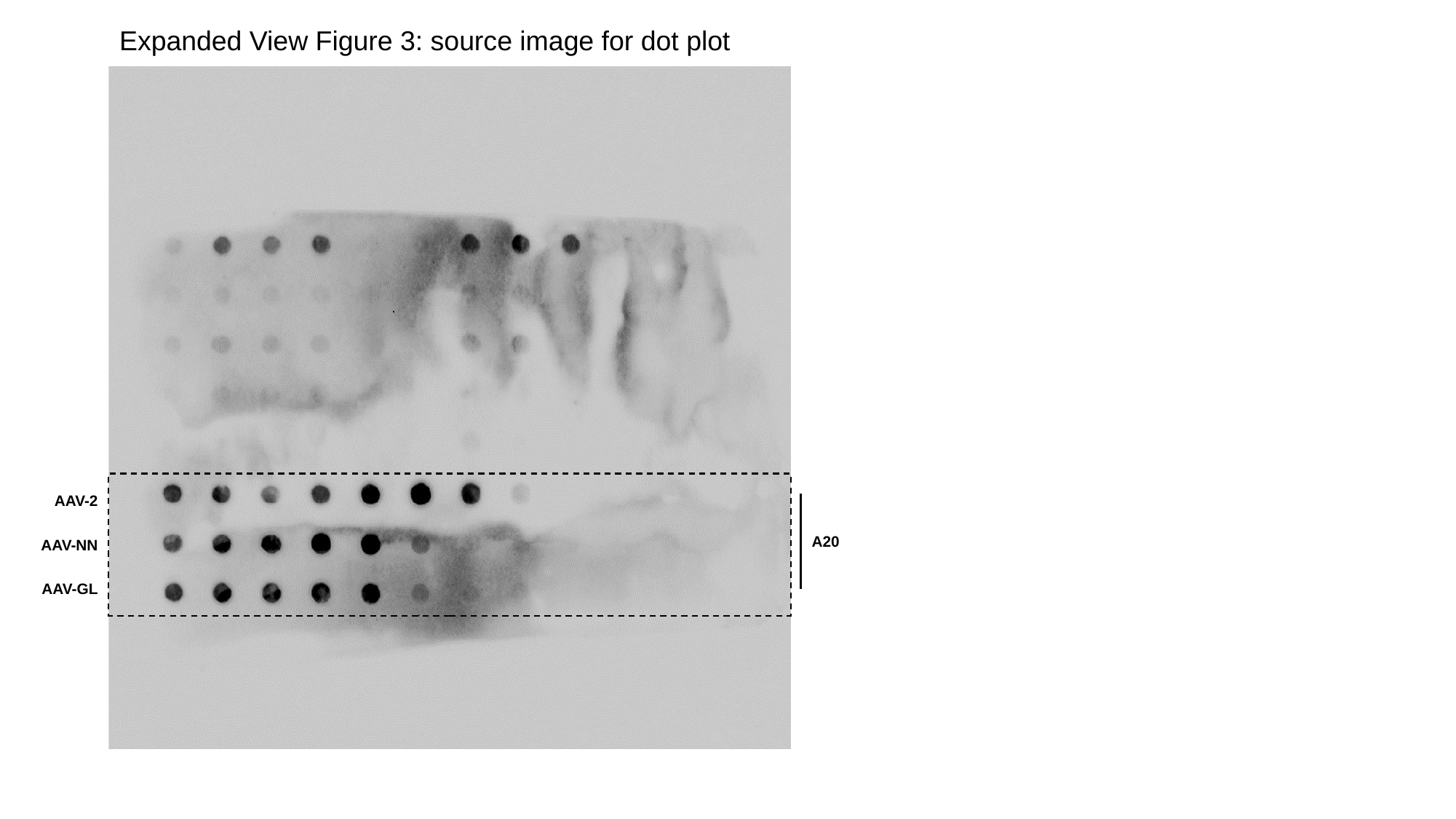

Expanded View Figure 3: source image for dot plot
AAV-2
AAV-NN
AAV-GL
A20

## Slide 2
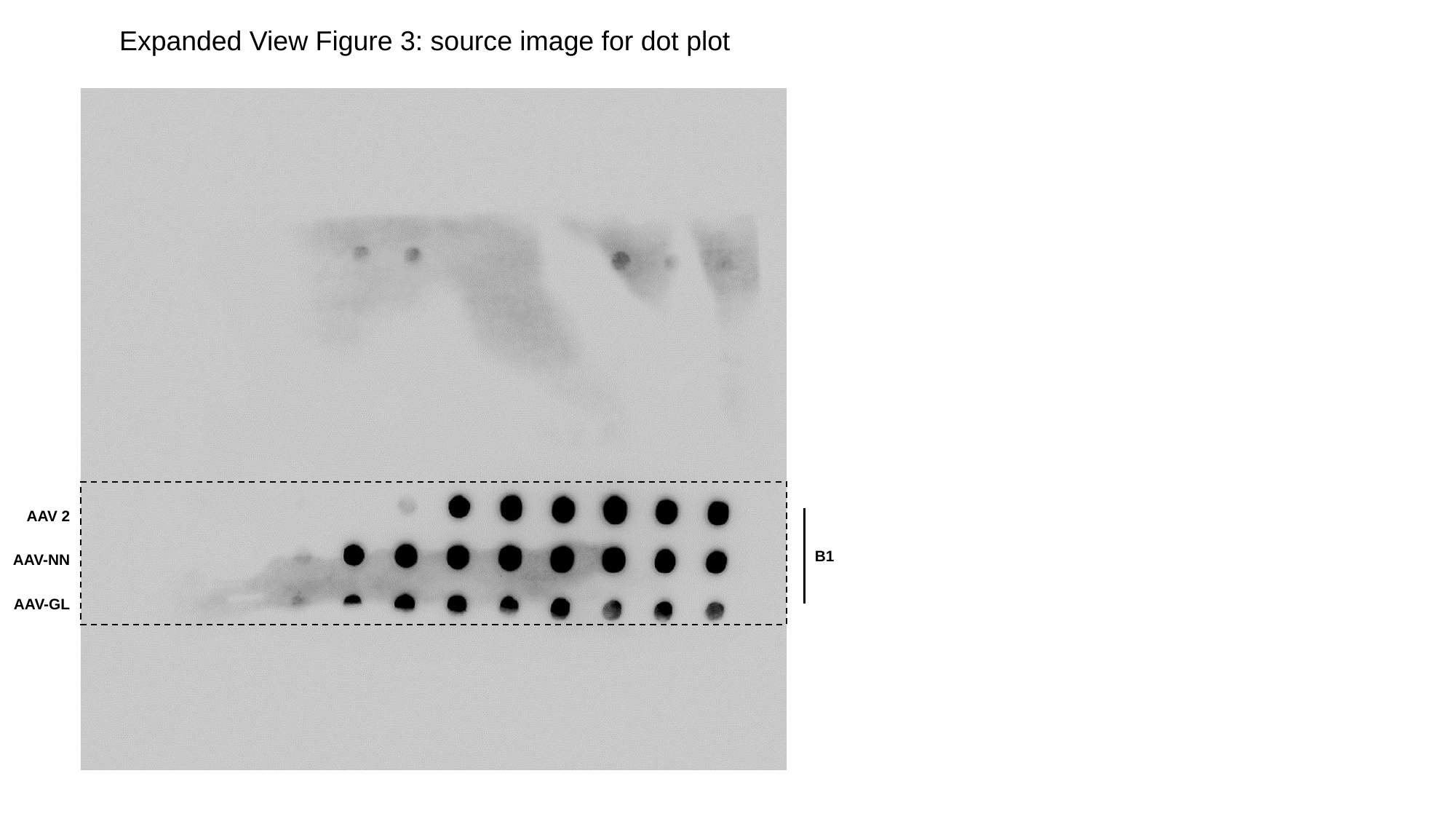

Expanded View Figure 3: source image for dot plot
AAV 2
AAV-NN
AAV-GL
B1
