# Supplementary figures and images for: Novel AAV capsids for intravitreal gene therapy of photoreceptor disorders
Source: EMBO Mol Med. 2021 Feb 22;13(4):e13392. doi: 10.15252/emmm.202013392 (PMC8033523; doi:10.15252/emmm.202013392)

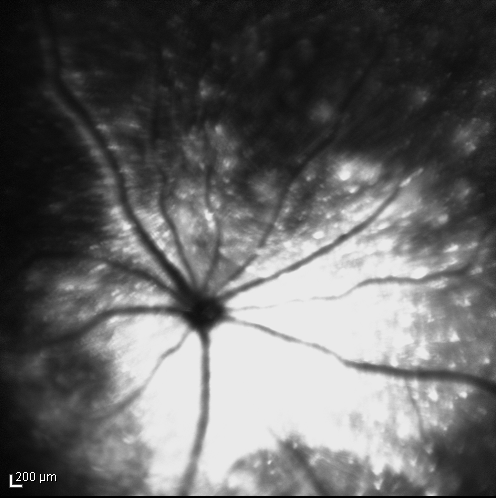

Supplement: Supplementary file 3 — Source Data for Expanded View [file EMMM-13-e13392-s008.zip › emmm202013392-sup-0011-SDataEVFig5/Source_Data_File_for_EV_Fig_5/14DPI_GL.tif]

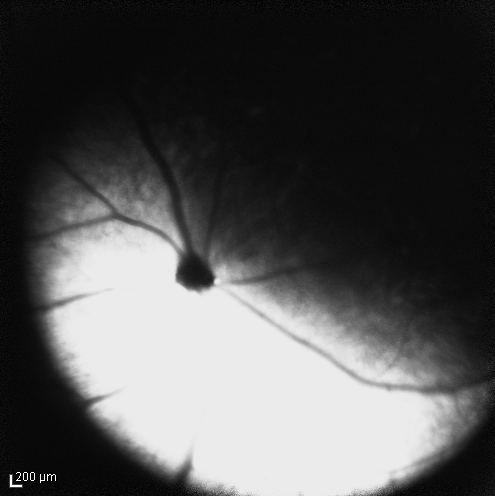

Supplement: Supplementary file 3 — Source Data for Expanded View [file EMMM-13-e13392-s008.zip › emmm202013392-sup-0011-SDataEVFig5/Source_Data_File_for_EV_Fig_5/14DPI_NN.tif]

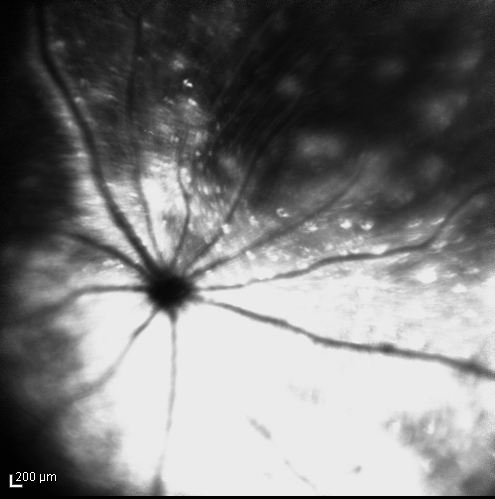

Supplement: Supplementary file 3 — Source Data for Expanded View [file EMMM-13-e13392-s008.zip › emmm202013392-sup-0011-SDataEVFig5/Source_Data_File_for_EV_Fig_5/21DPI_GL.tif]

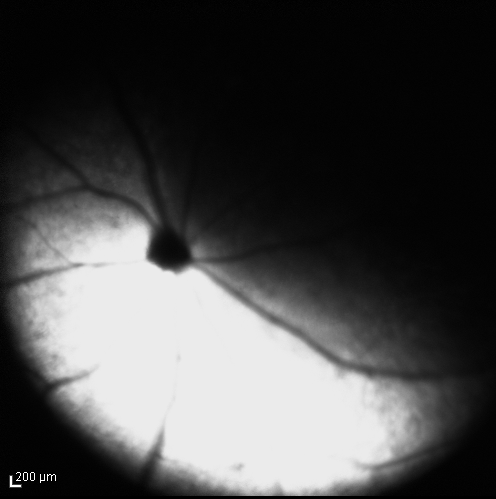

Supplement: Supplementary file 3 — Source Data for Expanded View [file EMMM-13-e13392-s008.zip › emmm202013392-sup-0011-SDataEVFig5/Source_Data_File_for_EV_Fig_5/21DPI_NN.tif]

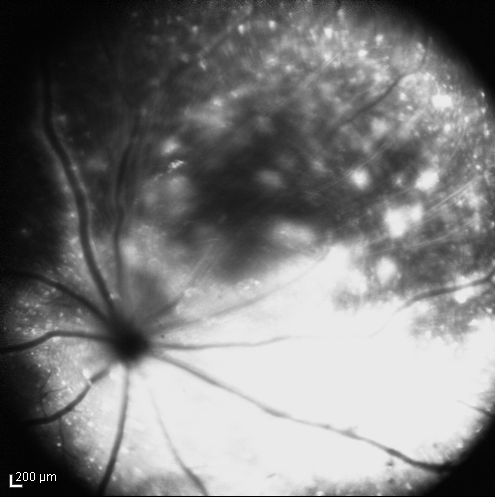

Supplement: Supplementary file 3 — Source Data for Expanded View [file EMMM-13-e13392-s008.zip › emmm202013392-sup-0011-SDataEVFig5/Source_Data_File_for_EV_Fig_5/29DPI_GL.tif]

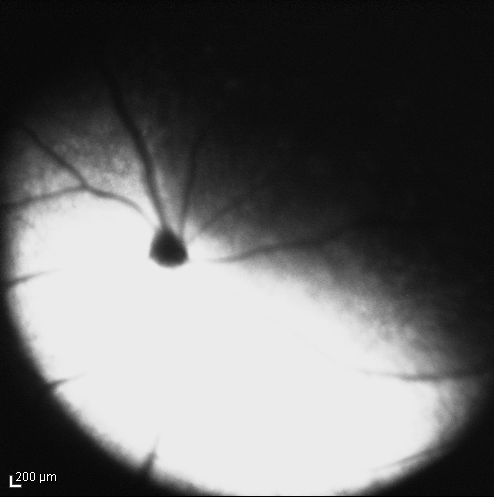

Supplement: Supplementary file 3 — Source Data for Expanded View [file EMMM-13-e13392-s008.zip › emmm202013392-sup-0011-SDataEVFig5/Source_Data_File_for_EV_Fig_5/29DPI_NN.tif]

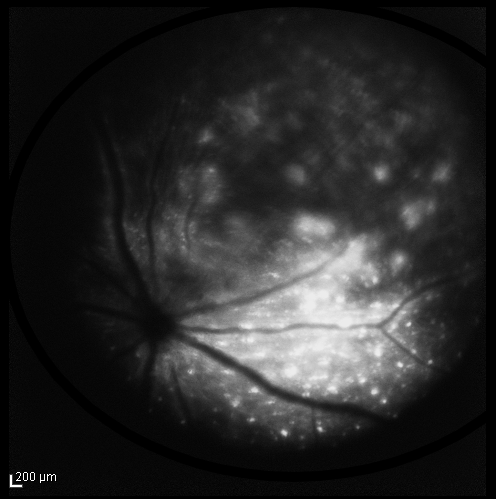

Supplement: Supplementary file 3 — Source Data for Expanded View [file EMMM-13-e13392-s008.zip › emmm202013392-sup-0011-SDataEVFig5/Source_Data_File_for_EV_Fig_5/7DPI_GL.tif]

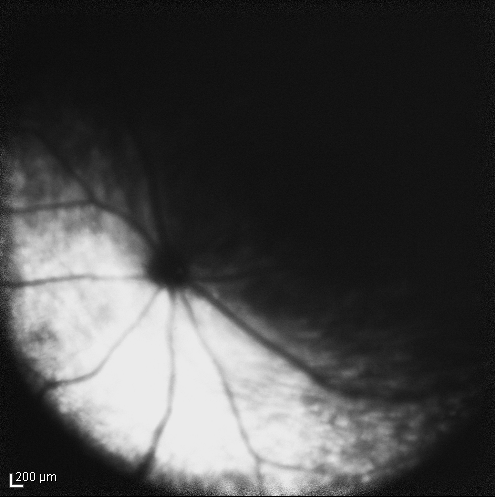

Supplement: Supplementary file 3 — Source Data for Expanded View [file EMMM-13-e13392-s008.zip › emmm202013392-sup-0011-SDataEVFig5/Source_Data_File_for_EV_Fig_5/7DPI_NN.tif]

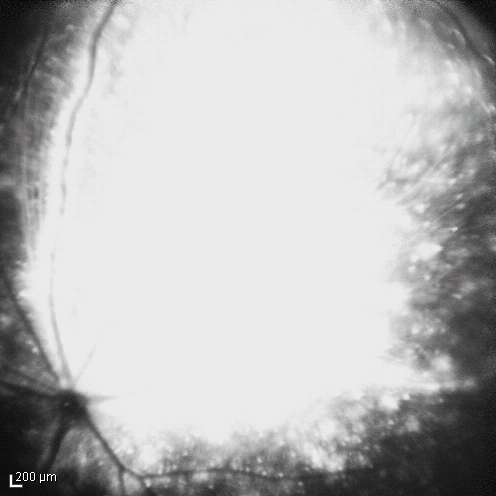

Supplement: Supplementary file 5 — Source Data for Figure 1 [file EMMM-13-e13392-s004.zip › Source_Data_File_for_Fig_1/7m8_1WPI_107.png]

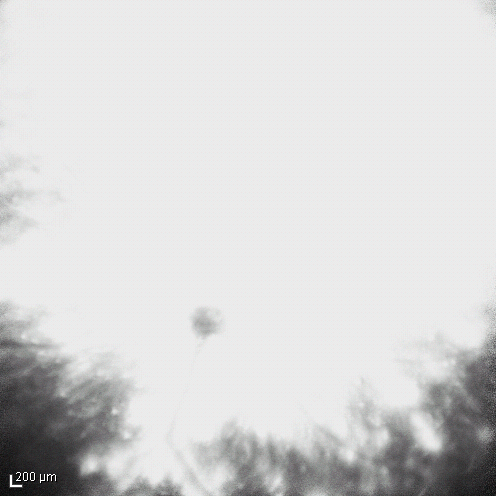

Supplement: Supplementary file 5 — Source Data for Figure 1 [file EMMM-13-e13392-s004.zip › Source_Data_File_for_Fig_1/7m8_2WPI_107.png]

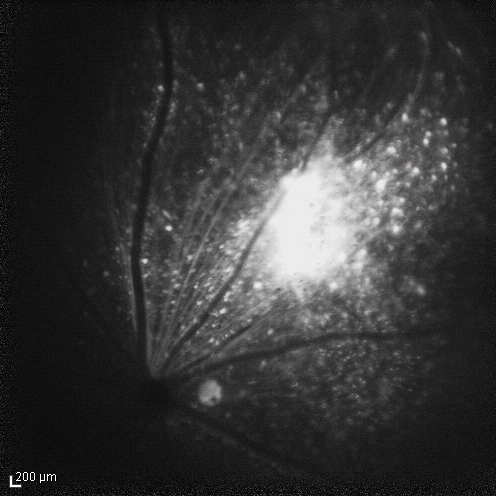

Supplement: Supplementary file 5 — Source Data for Figure 1 [file EMMM-13-e13392-s004.zip › Source_Data_File_for_Fig_1/AAV2_1WPI_107.png]

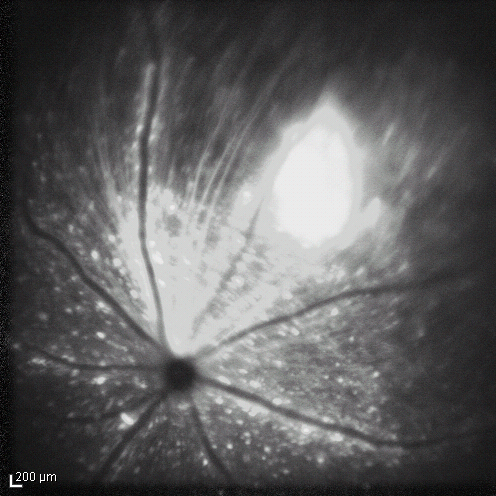

Supplement: Supplementary file 5 — Source Data for Figure 1 [file EMMM-13-e13392-s004.zip › Source_Data_File_for_Fig_1/AAV2_2WPI_107.png]

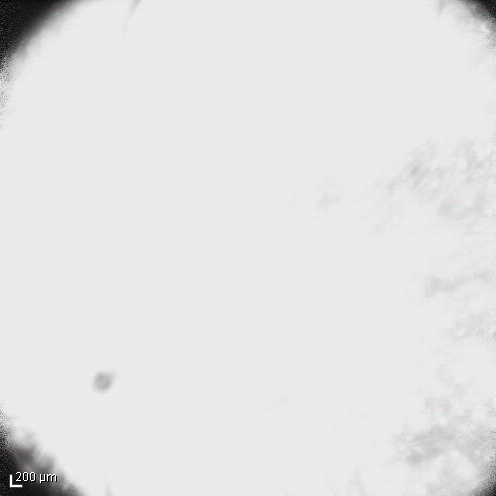

Supplement: Supplementary file 5 — Source Data for Figure 1 [file EMMM-13-e13392-s004.zip › Source_Data_File_for_Fig_1/GL_1WPI_107.png]

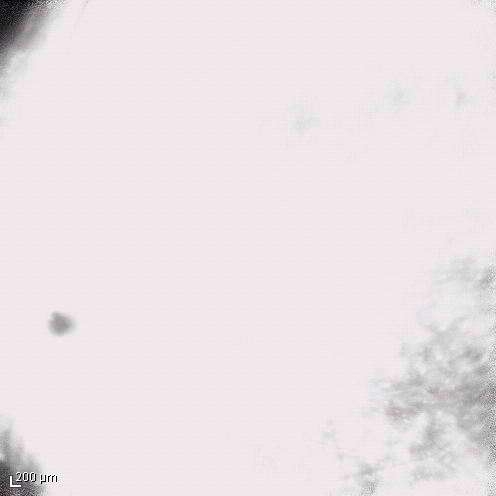

Supplement: Supplementary file 5 — Source Data for Figure 1 [file EMMM-13-e13392-s004.zip › Source_Data_File_for_Fig_1/GL_2WPI_107.png]

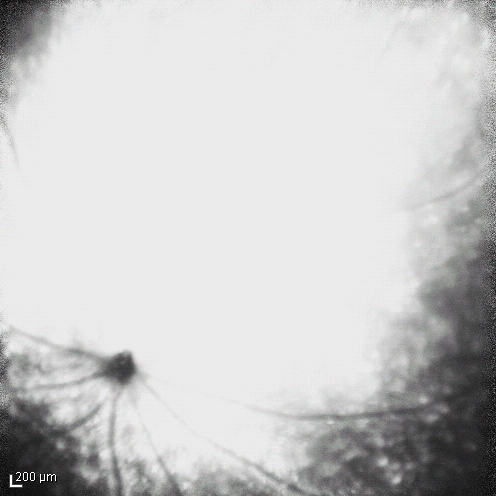

Supplement: Supplementary file 5 — Source Data for Figure 1 [file EMMM-13-e13392-s004.zip › Source_Data_File_for_Fig_1/NN_1WPI_107.png]

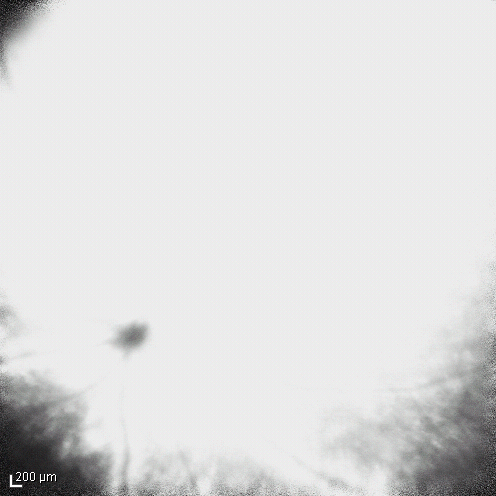

Supplement: Supplementary file 5 — Source Data for Figure 1 [file EMMM-13-e13392-s004.zip › Source_Data_File_for_Fig_1/NN_2WPI_107.png]

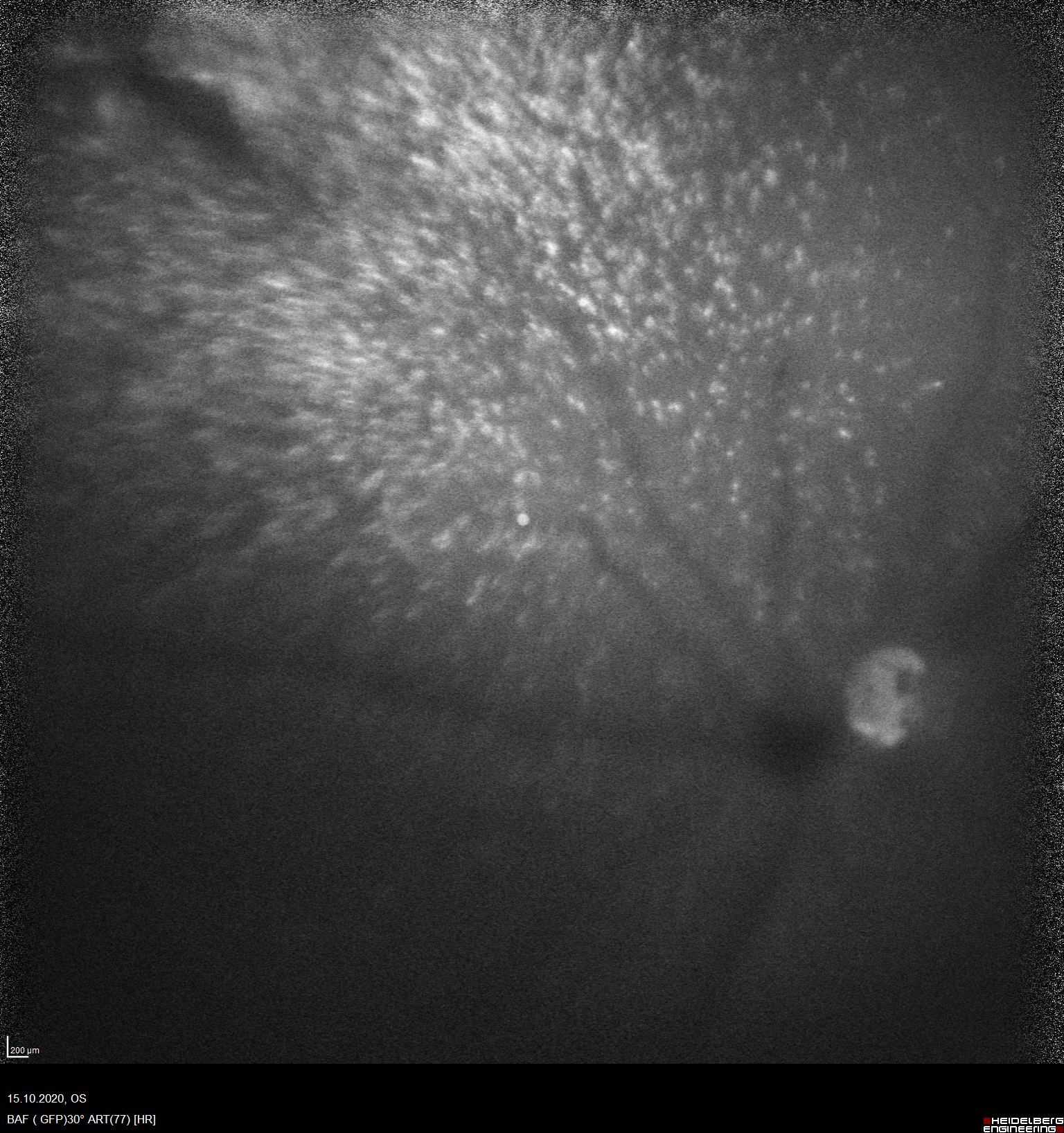

Supplement: Supplementary file 6 — Source Data for Figure 2 [file EMMM-13-e13392-s001.zip › Source_Data_File_for_Fig_2/1WPI_AAV2.7m8.tif]

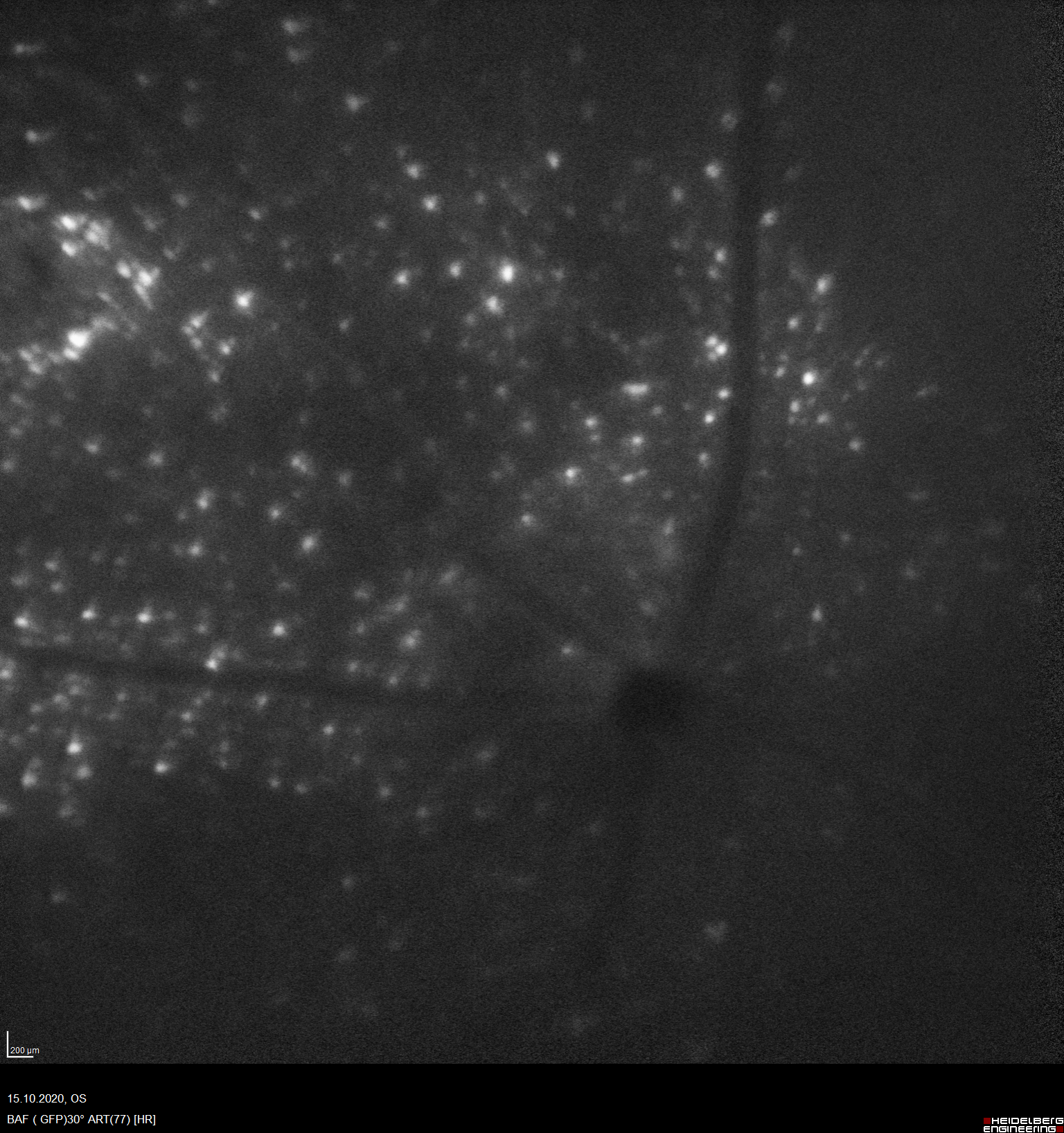

Supplement: Supplementary file 6 — Source Data for Figure 2 [file EMMM-13-e13392-s001.zip › Source_Data_File_for_Fig_2/1WPI_AAV2.GL.tif]

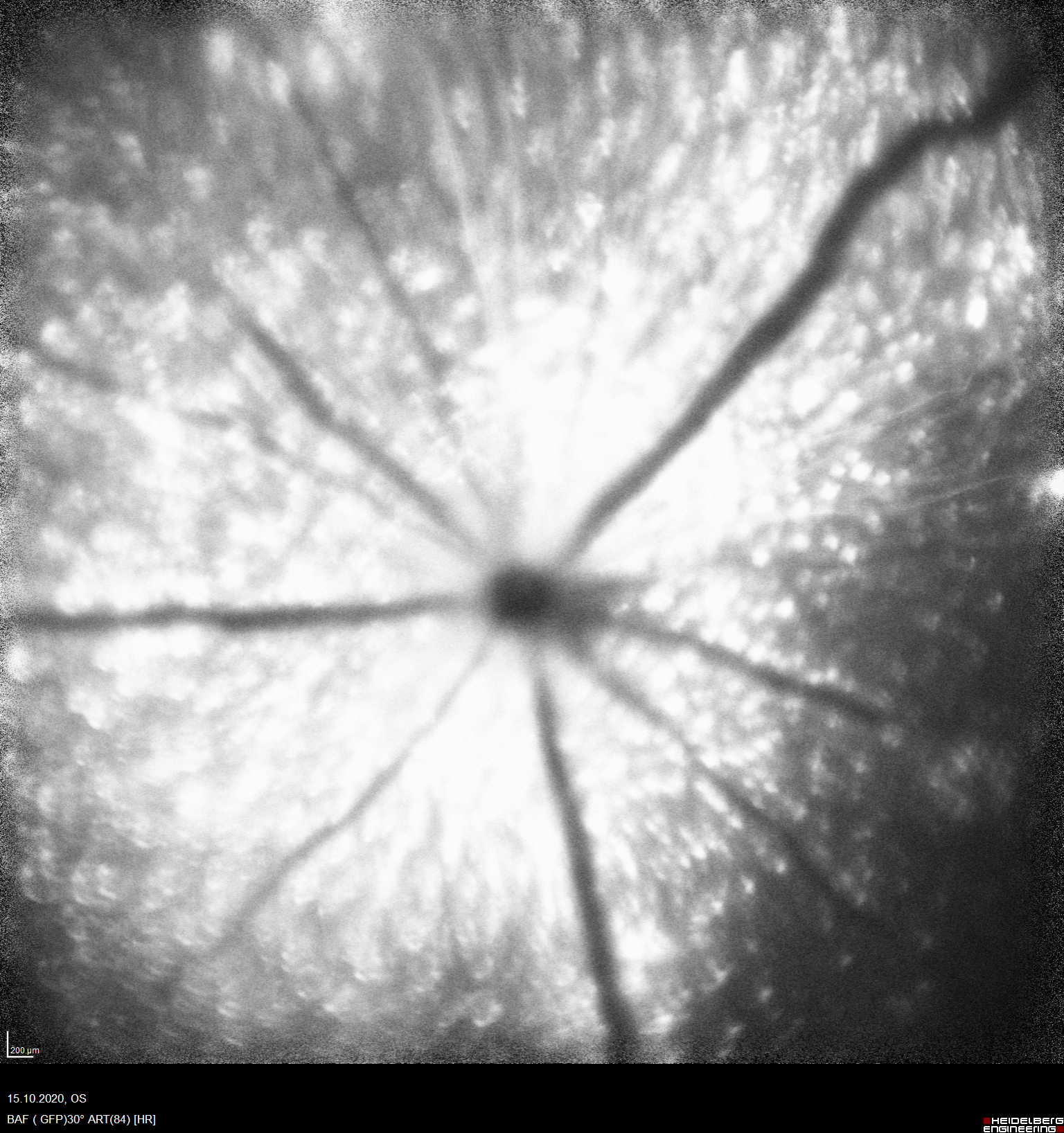

Supplement: Supplementary file 6 — Source Data for Figure 2 [file EMMM-13-e13392-s001.zip › Source_Data_File_for_Fig_2/1WPI_AAV2.NN.tif]

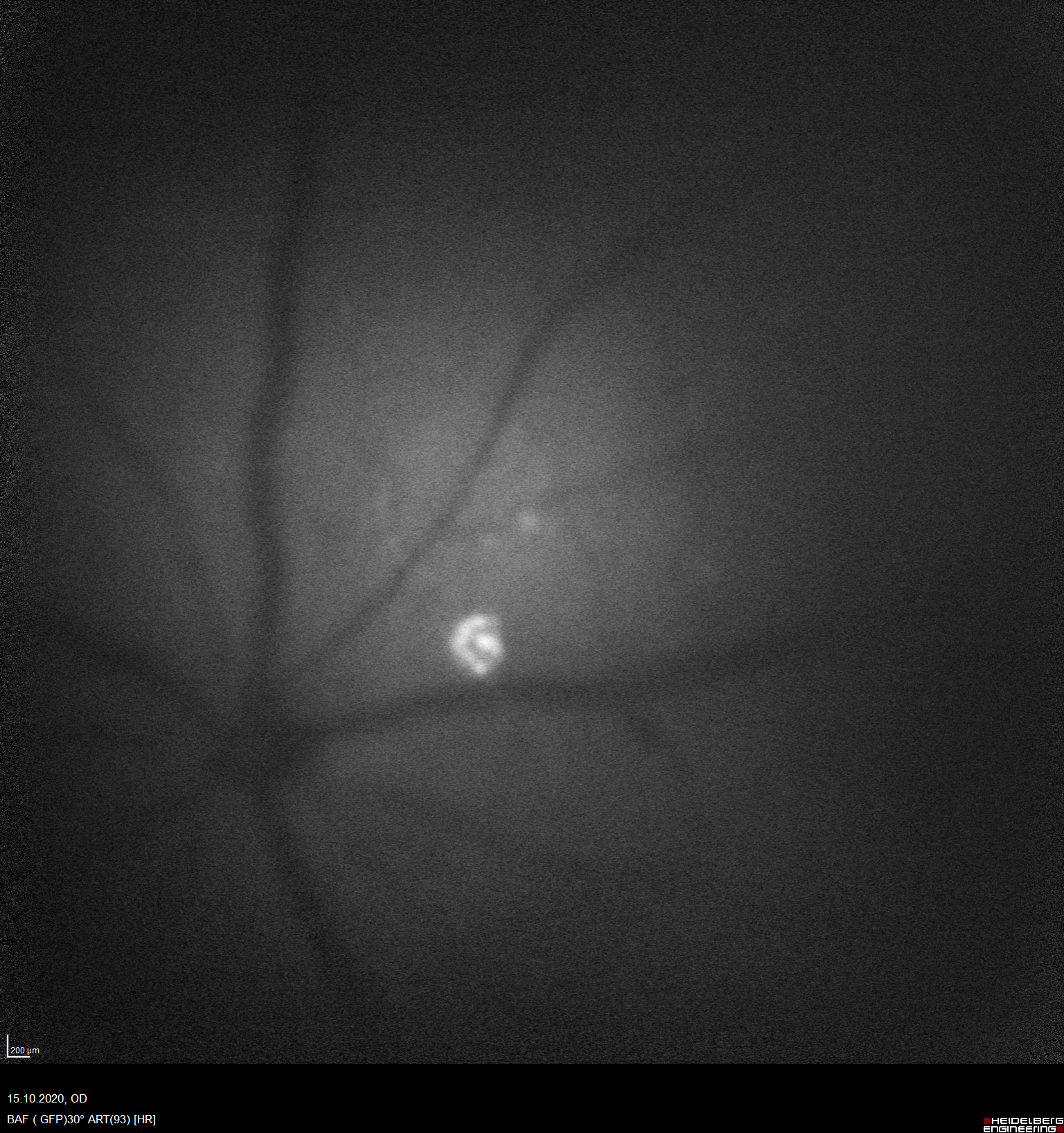

Supplement: Supplementary file 6 — Source Data for Figure 2 [file EMMM-13-e13392-s001.zip › Source_Data_File_for_Fig_2/1WPI_AAV2.tif]

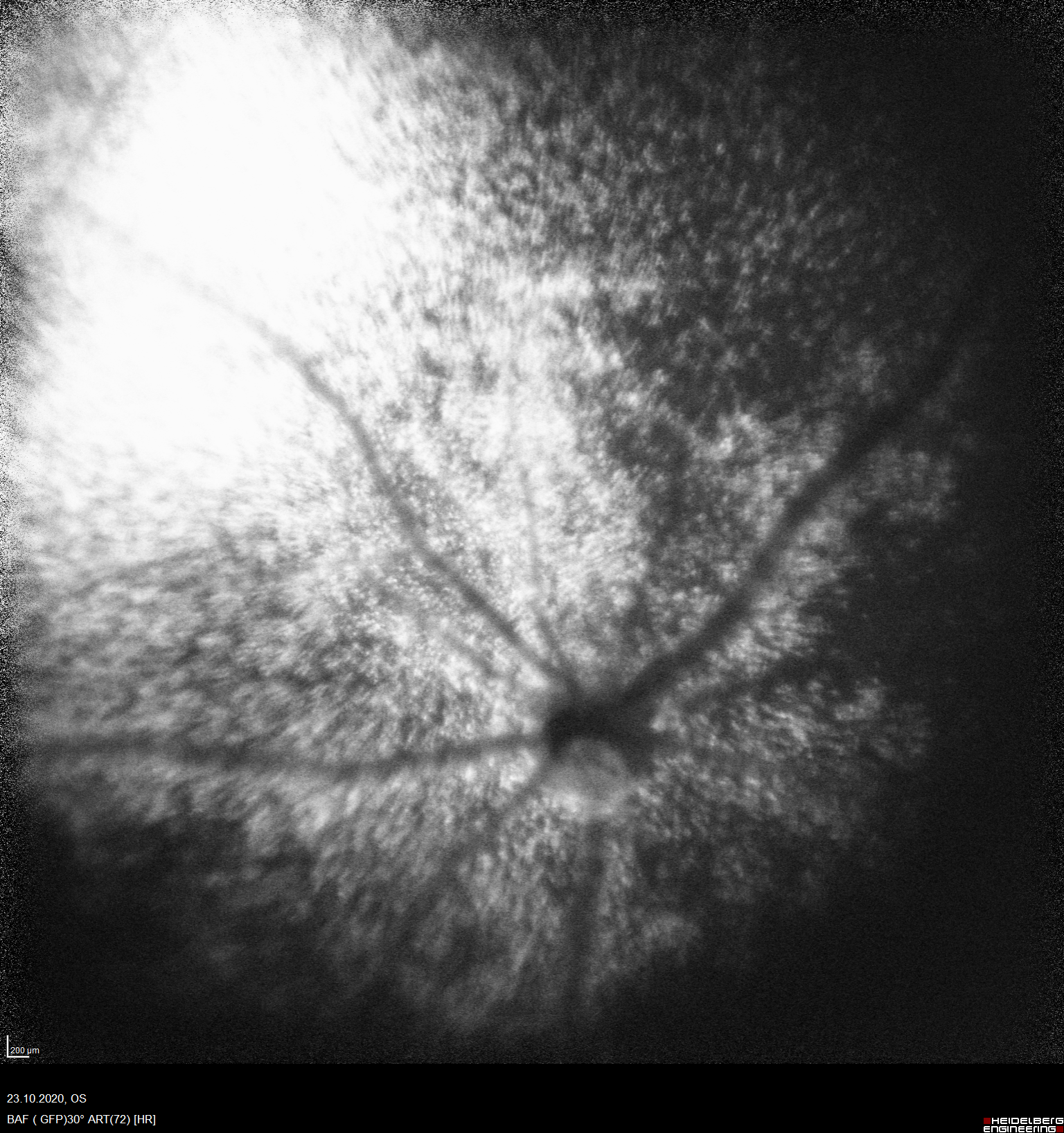

Supplement: Supplementary file 6 — Source Data for Figure 2 [file EMMM-13-e13392-s001.zip › Source_Data_File_for_Fig_2/2WPI_AAV2.7m8.tif]

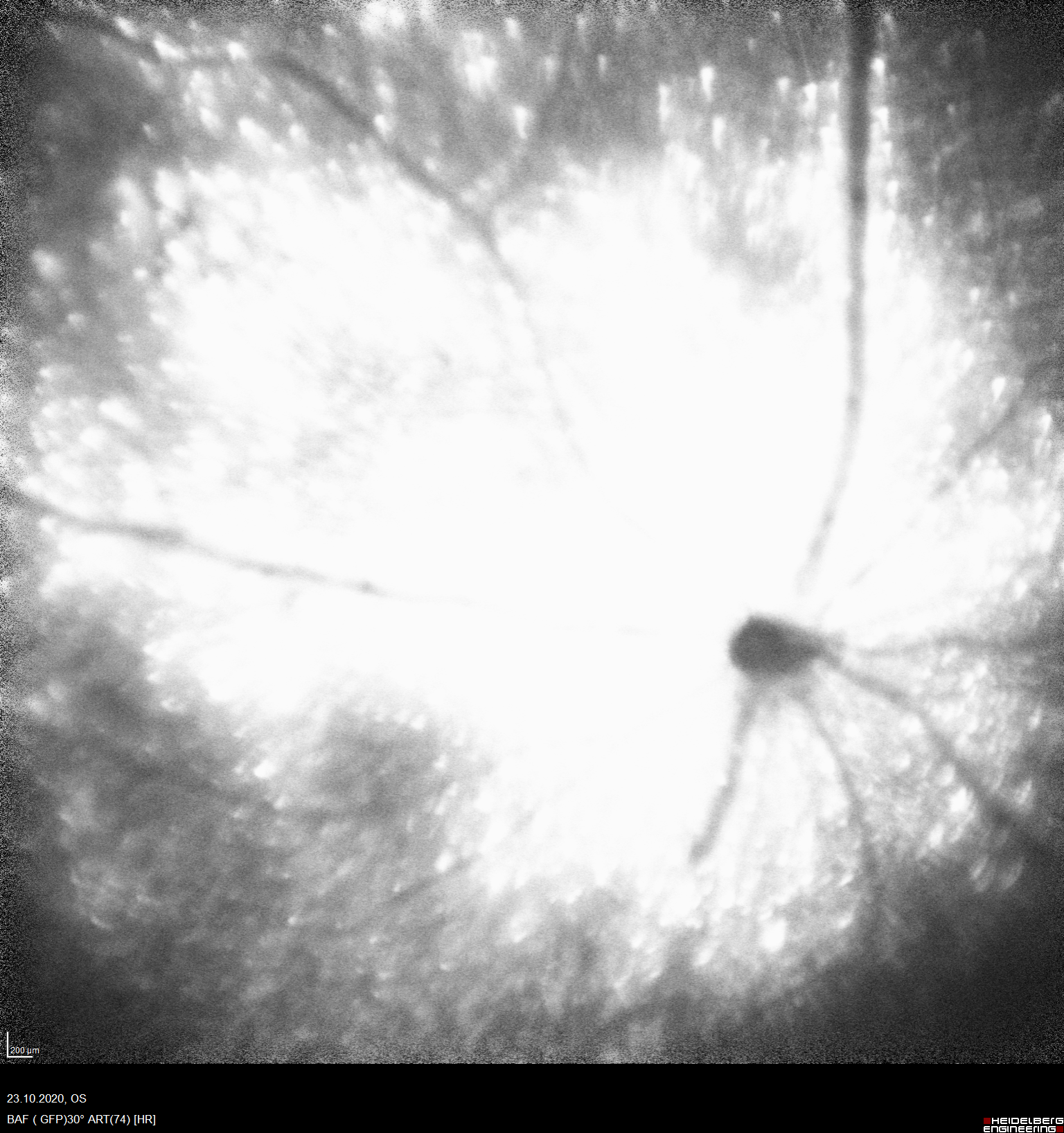

Supplement: Supplementary file 6 — Source Data for Figure 2 [file EMMM-13-e13392-s001.zip › Source_Data_File_for_Fig_2/2WPI_AAV2.GL.tif]

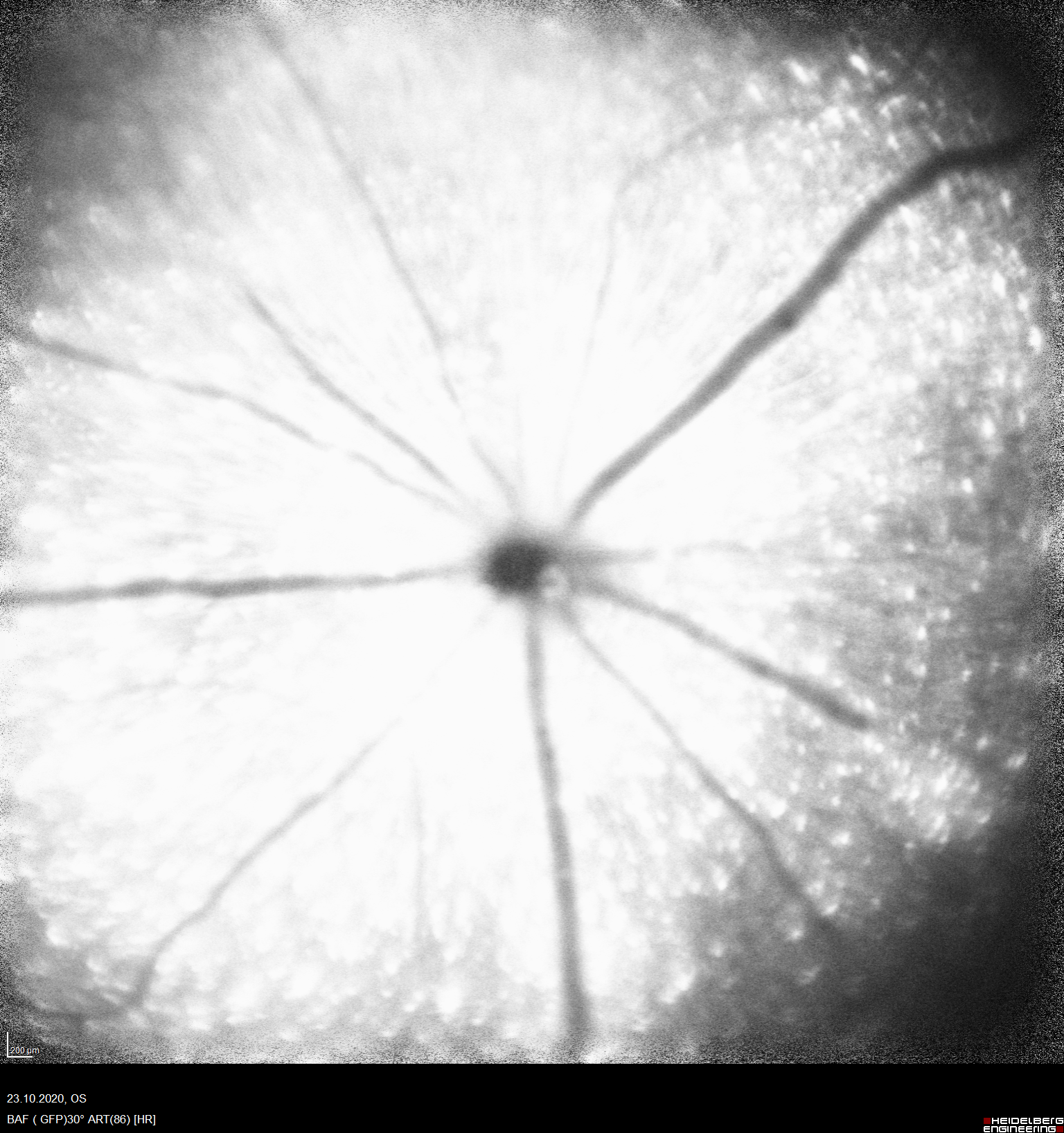

Supplement: Supplementary file 6 — Source Data for Figure 2 [file EMMM-13-e13392-s001.zip › Source_Data_File_for_Fig_2/2WPI_AAV2.NN.tif]

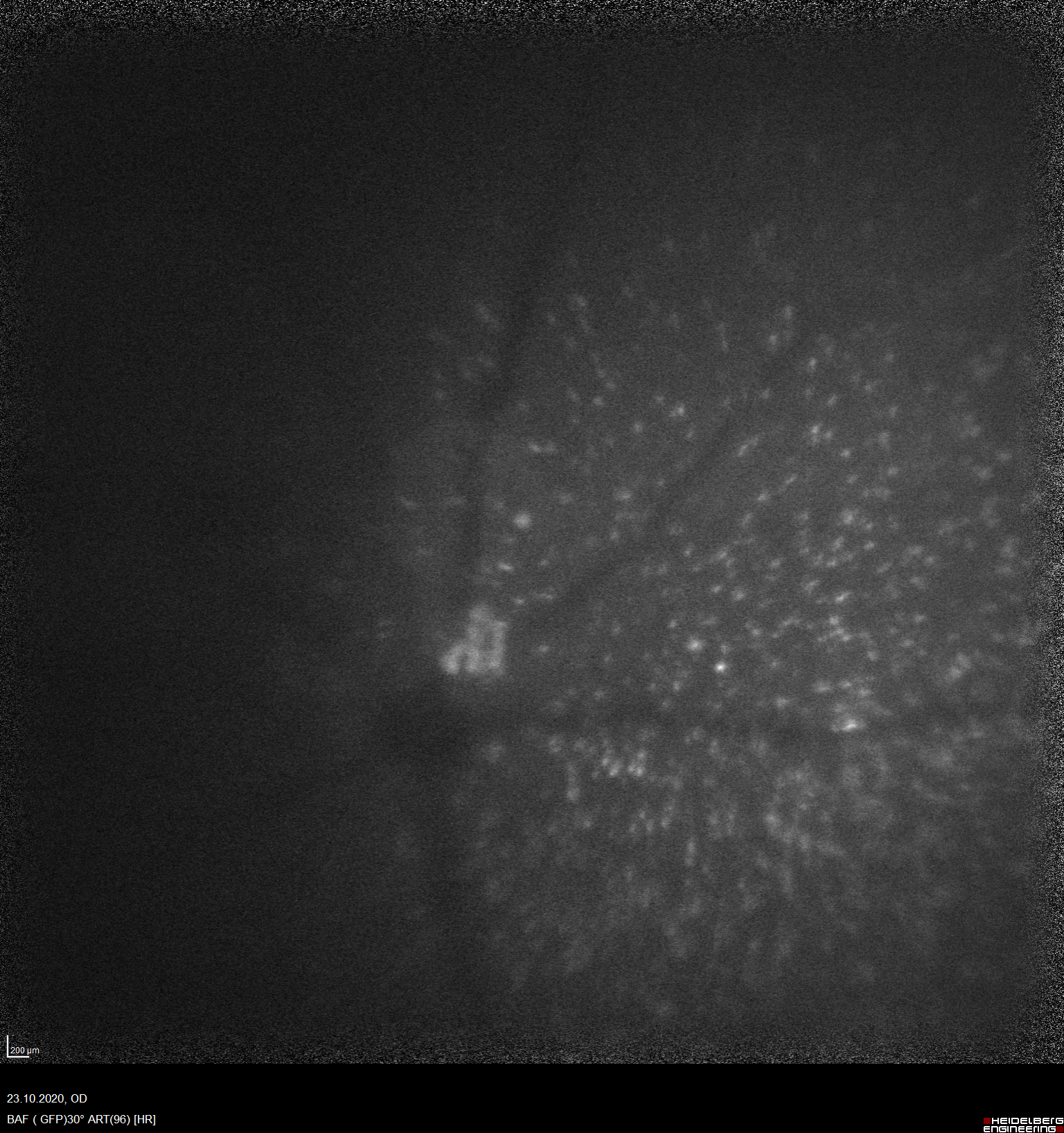

Supplement: Supplementary file 6 — Source Data for Figure 2 [file EMMM-13-e13392-s001.zip › Source_Data_File_for_Fig_2/2WPI_AAV2.tif]

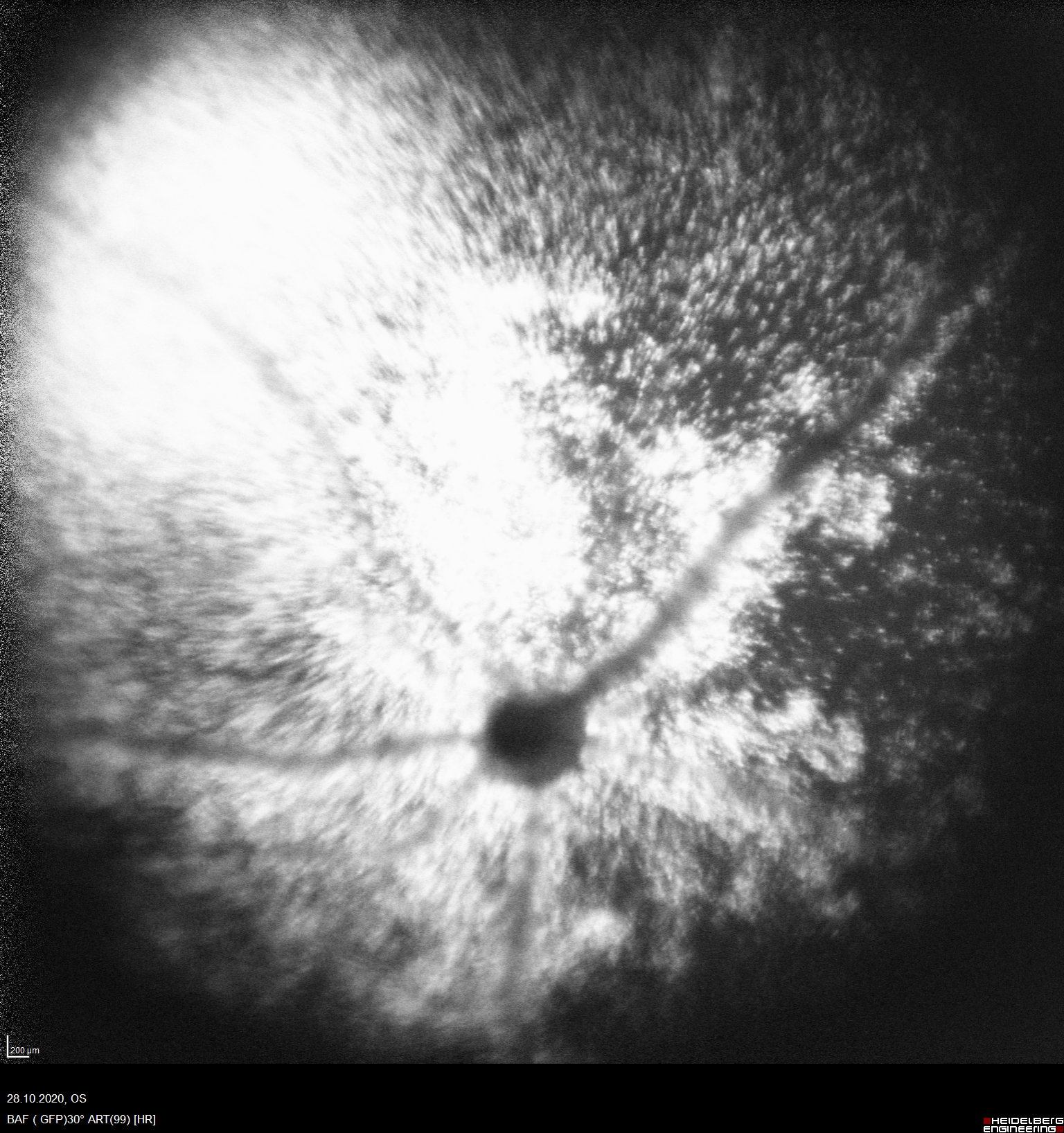

Supplement: Supplementary file 6 — Source Data for Figure 2 [file EMMM-13-e13392-s001.zip › Source_Data_File_for_Fig_2/3WPI_AAV2.7m8.tif]

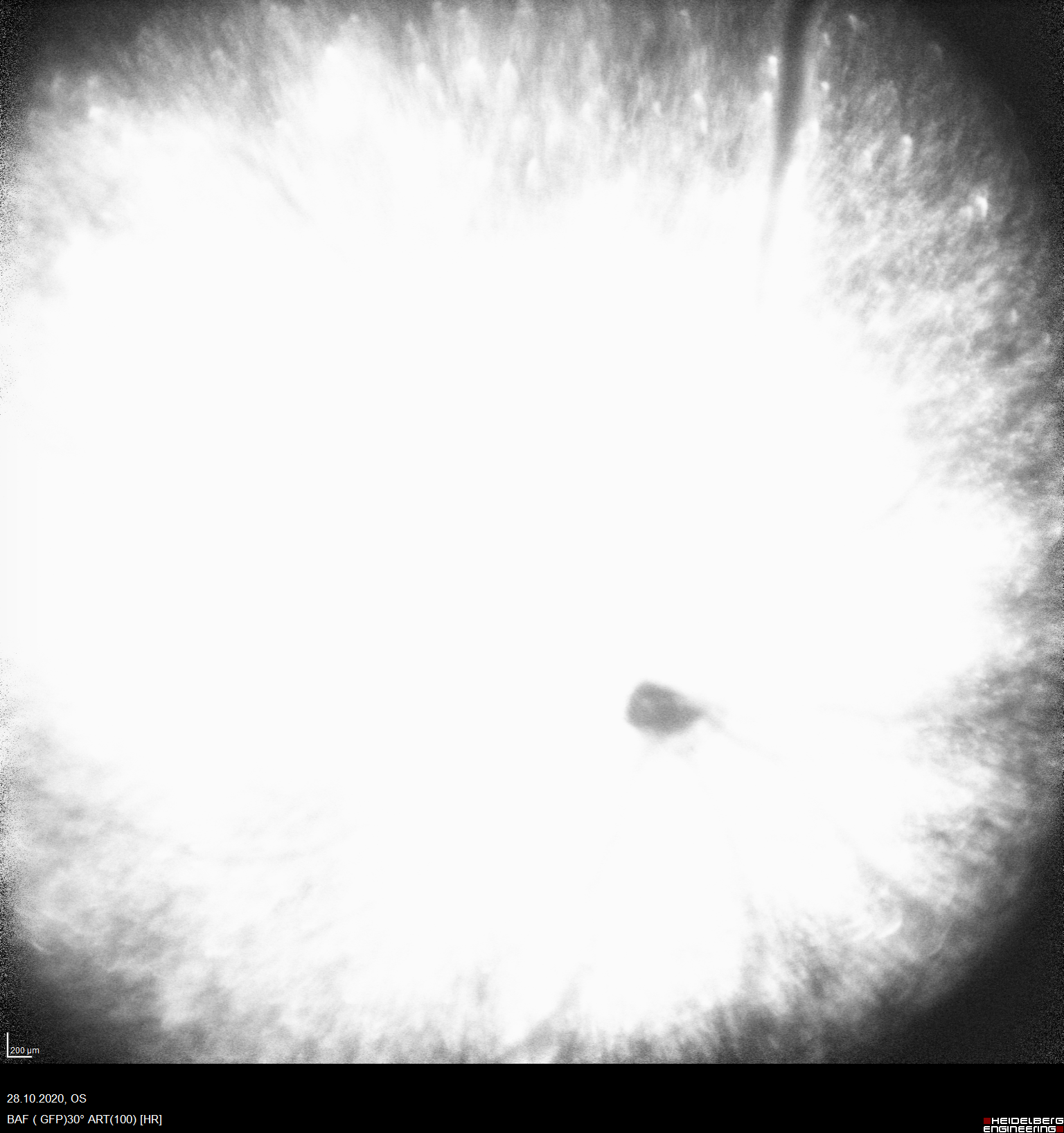

Supplement: Supplementary file 6 — Source Data for Figure 2 [file EMMM-13-e13392-s001.zip › Source_Data_File_for_Fig_2/3WPI_AAV2.GL.tif]

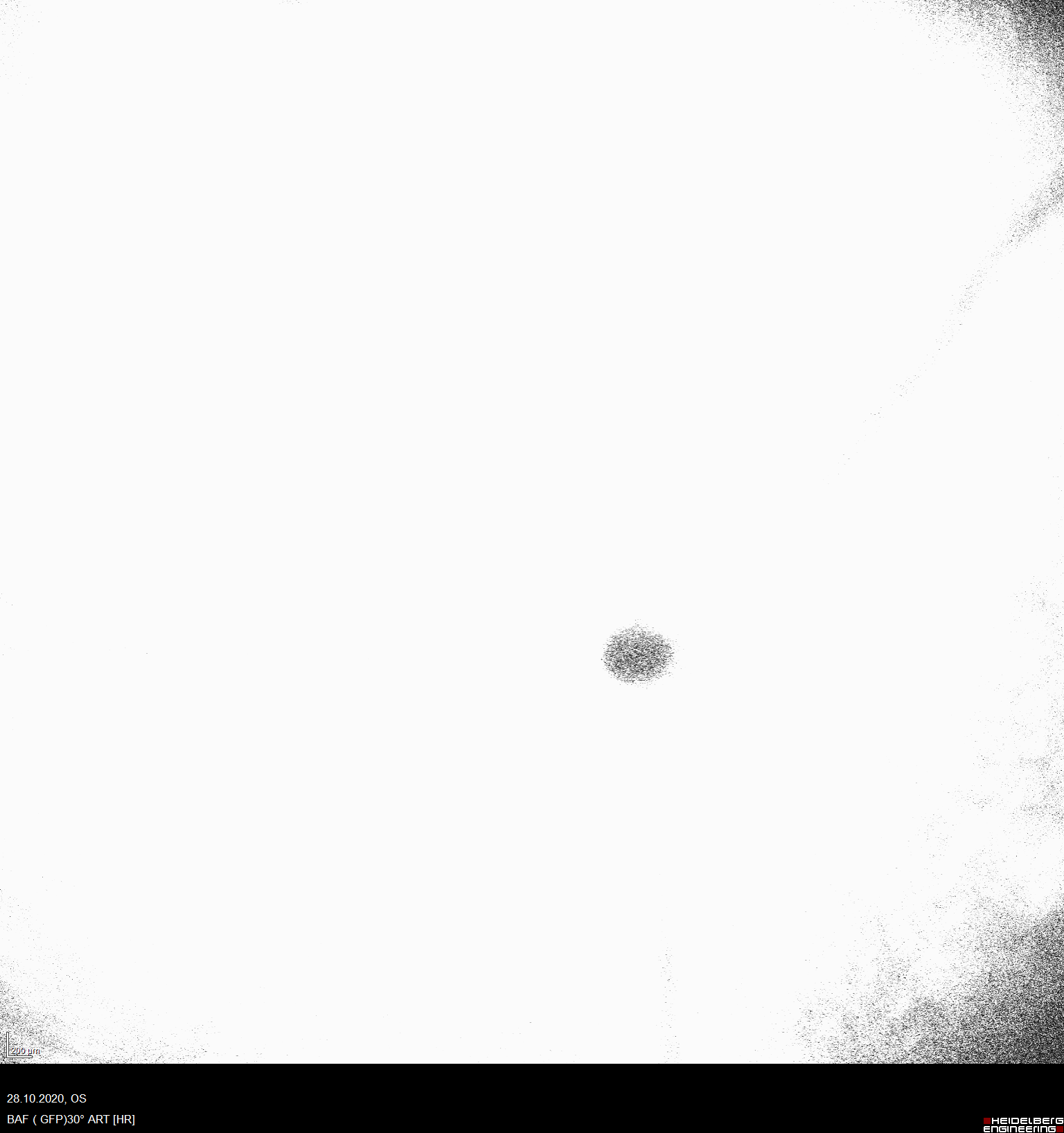

Supplement: Supplementary file 6 — Source Data for Figure 2 [file EMMM-13-e13392-s001.zip › Source_Data_File_for_Fig_2/3WPI_AAV2.NN.tif]

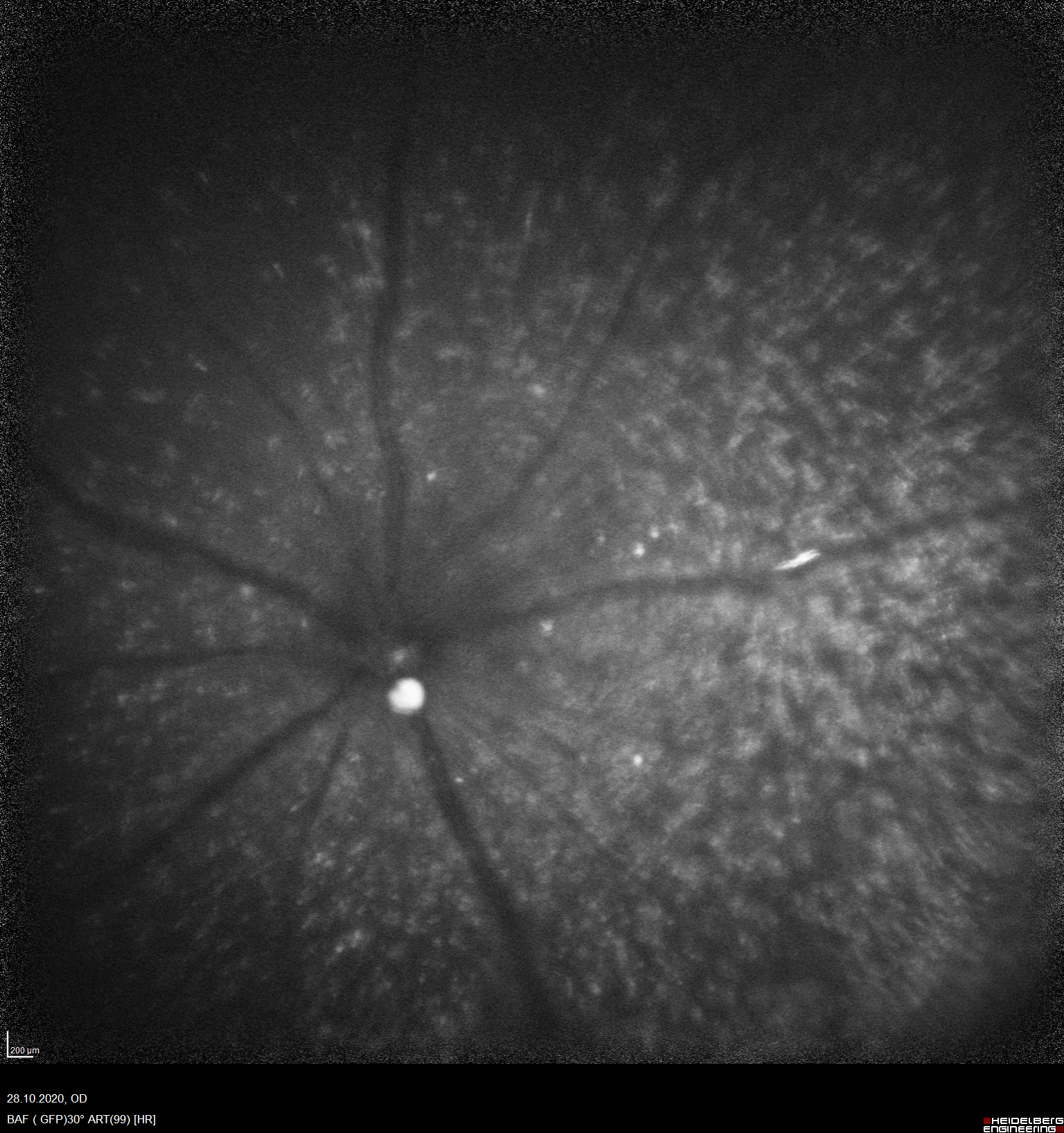

Supplement: Supplementary file 6 — Source Data for Figure 2 [file EMMM-13-e13392-s001.zip › Source_Data_File_for_Fig_2/3WPI_AAV2.tif]

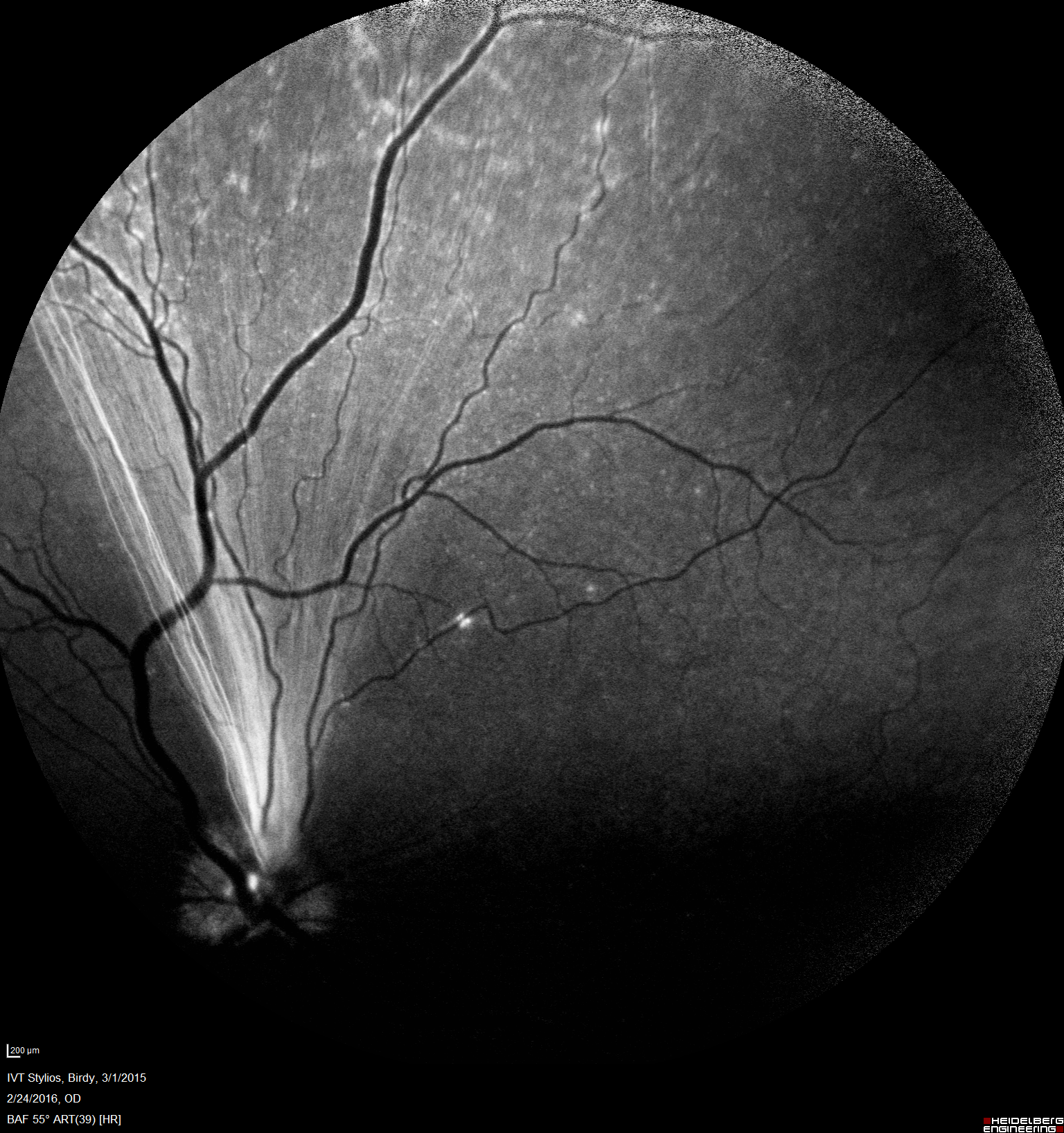

Supplement: Supplementary file 7 — Source Data for Figure 3 [file EMMM-13-e13392-s005.zip › Source_Data_File_for_Fig_3/4WPI_AAV2.tif]

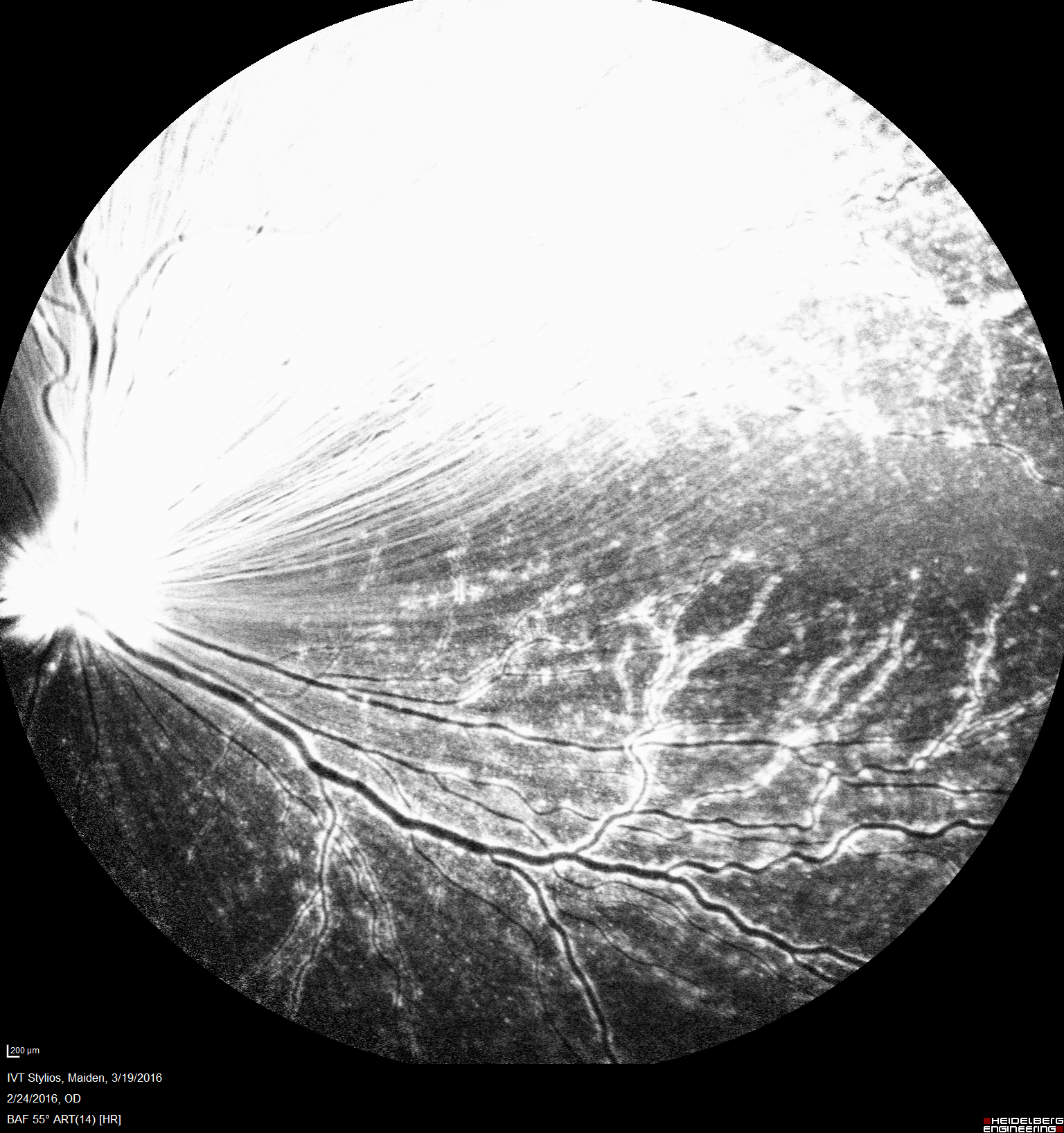

Supplement: Supplementary file 7 — Source Data for Figure 3 [file EMMM-13-e13392-s005.zip › Source_Data_File_for_Fig_3/4WPI_GL.tif]

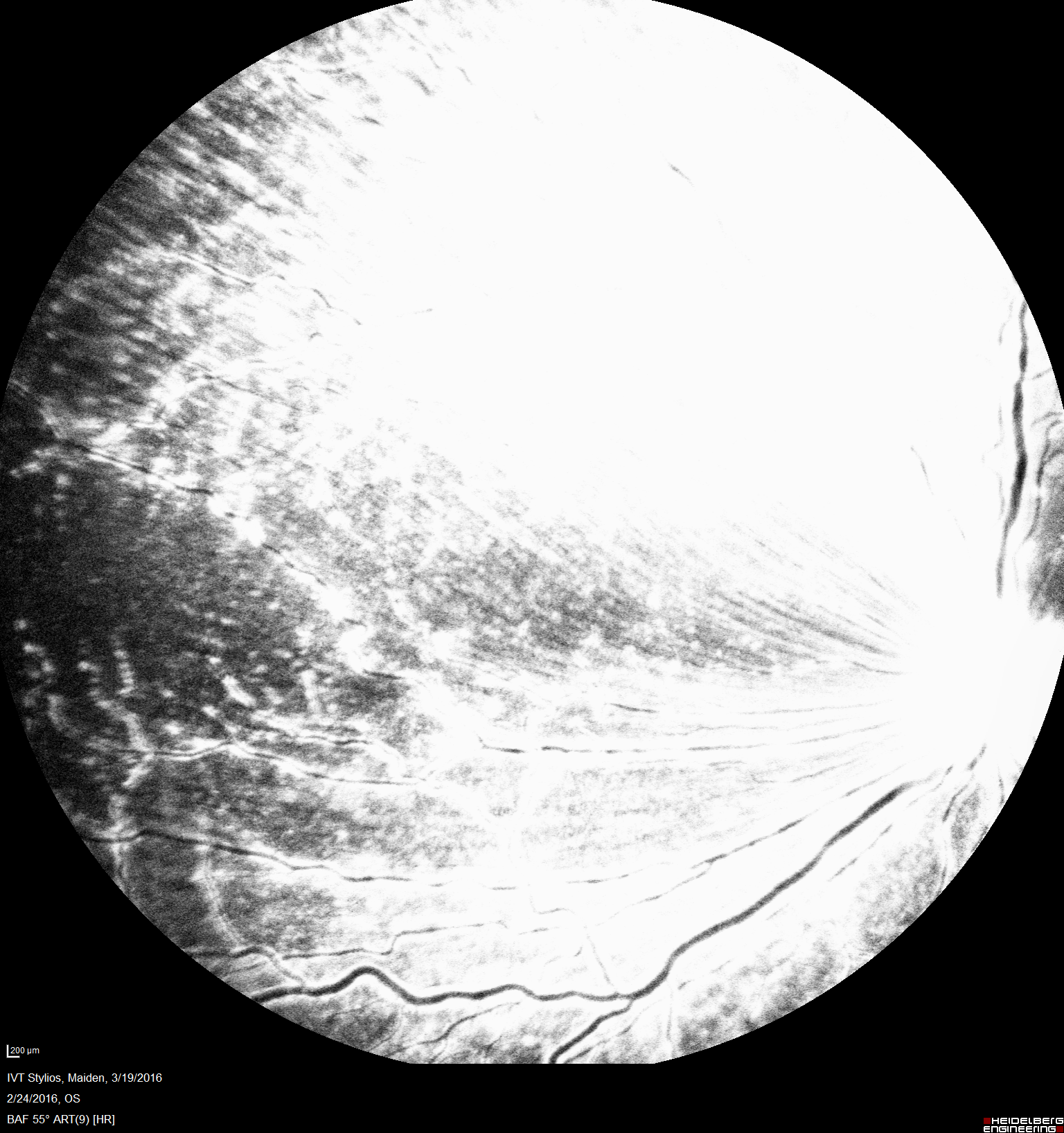

Supplement: Supplementary file 7 — Source Data for Figure 3 [file EMMM-13-e13392-s005.zip › Source_Data_File_for_Fig_3/4WPI_NN.tif]

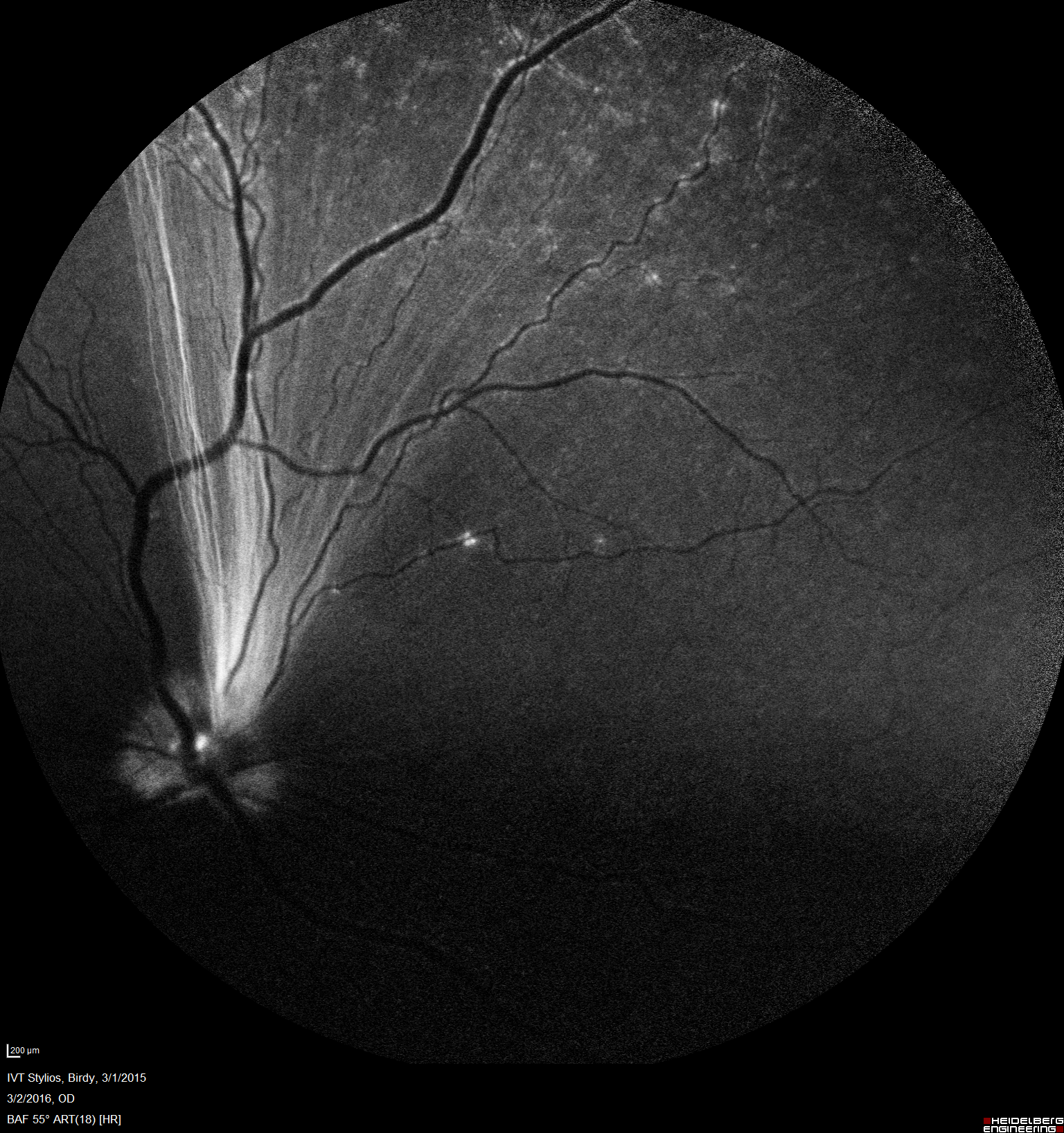

Supplement: Supplementary file 7 — Source Data for Figure 3 [file EMMM-13-e13392-s005.zip › Source_Data_File_for_Fig_3/5WPI_AAV2.tif]

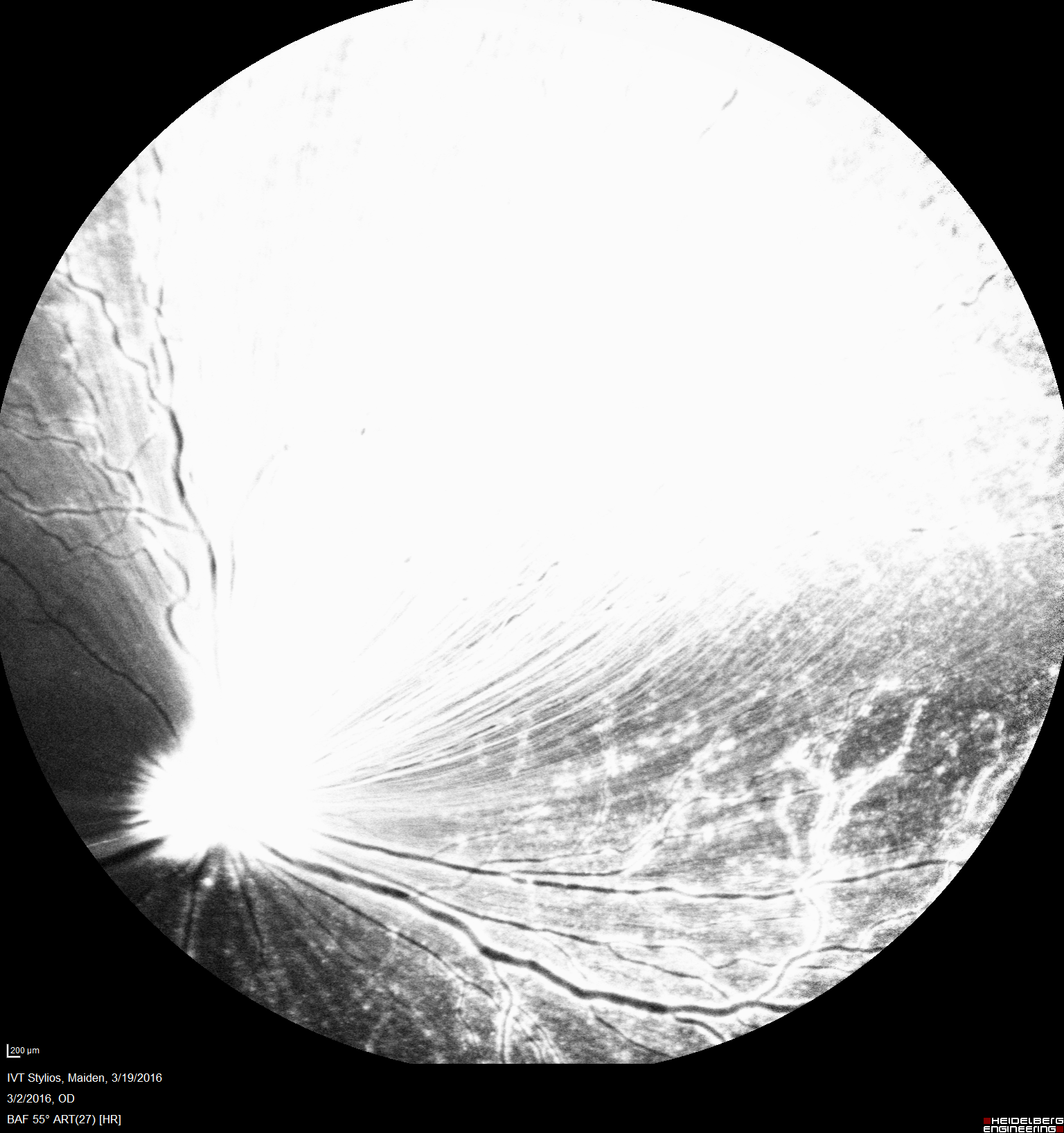

Supplement: Supplementary file 7 — Source Data for Figure 3 [file EMMM-13-e13392-s005.zip › Source_Data_File_for_Fig_3/5WPI_GL.tif]

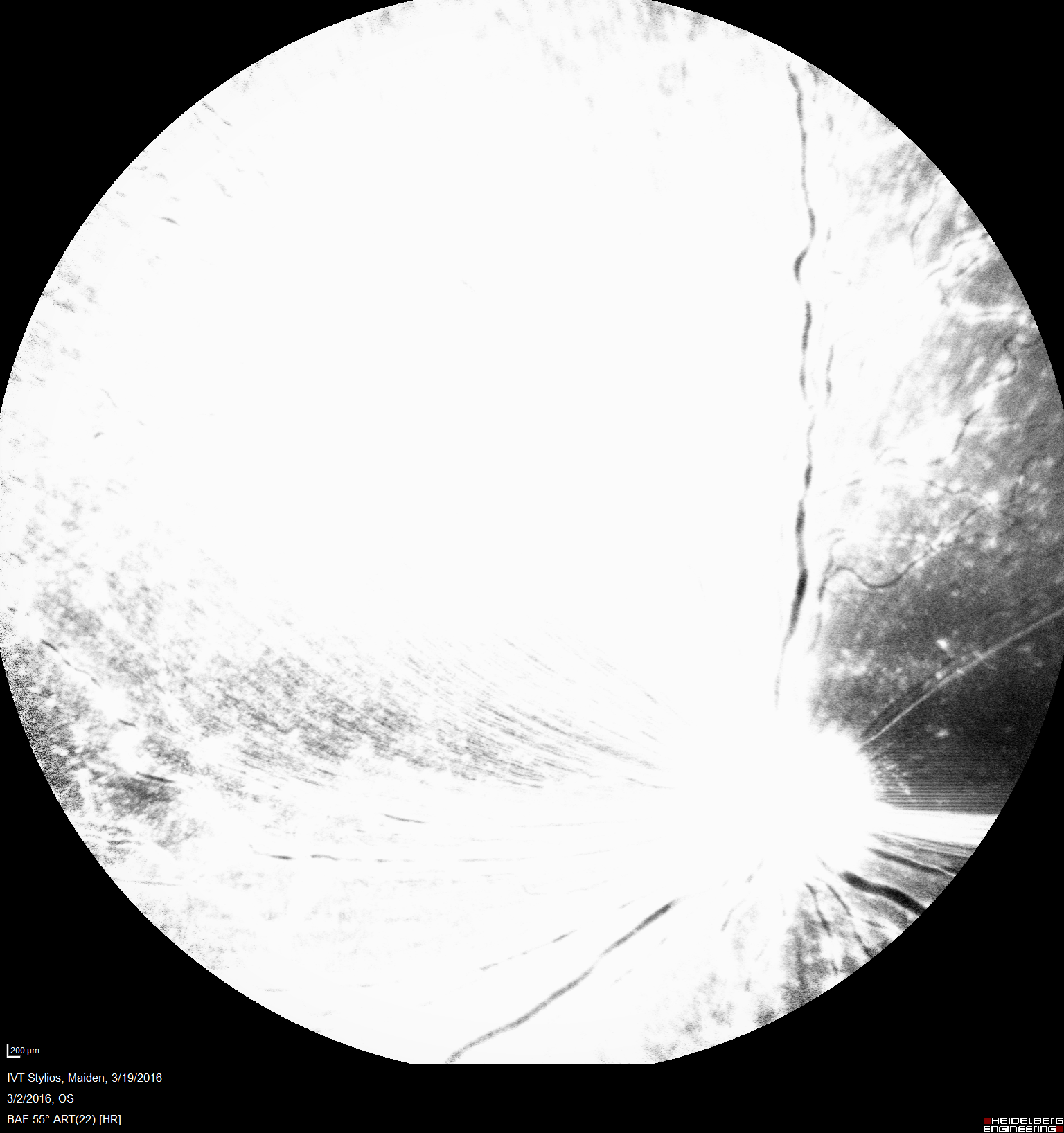

Supplement: Supplementary file 7 — Source Data for Figure 3 [file EMMM-13-e13392-s005.zip › Source_Data_File_for_Fig_3/5WPI_NN.tif]

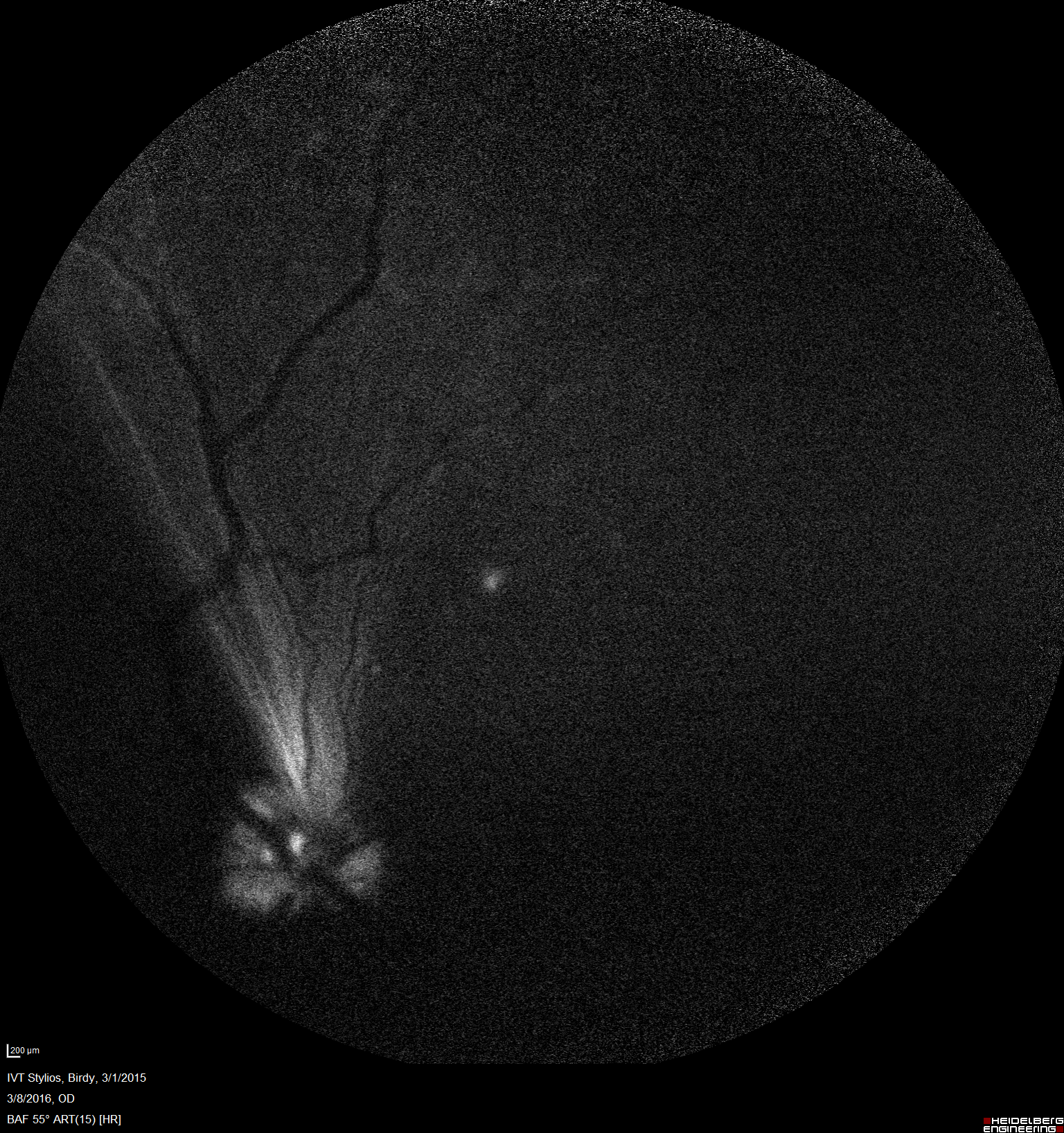

Supplement: Supplementary file 7 — Source Data for Figure 3 [file EMMM-13-e13392-s005.zip › Source_Data_File_for_Fig_3/6WPI_AAV2.tif]

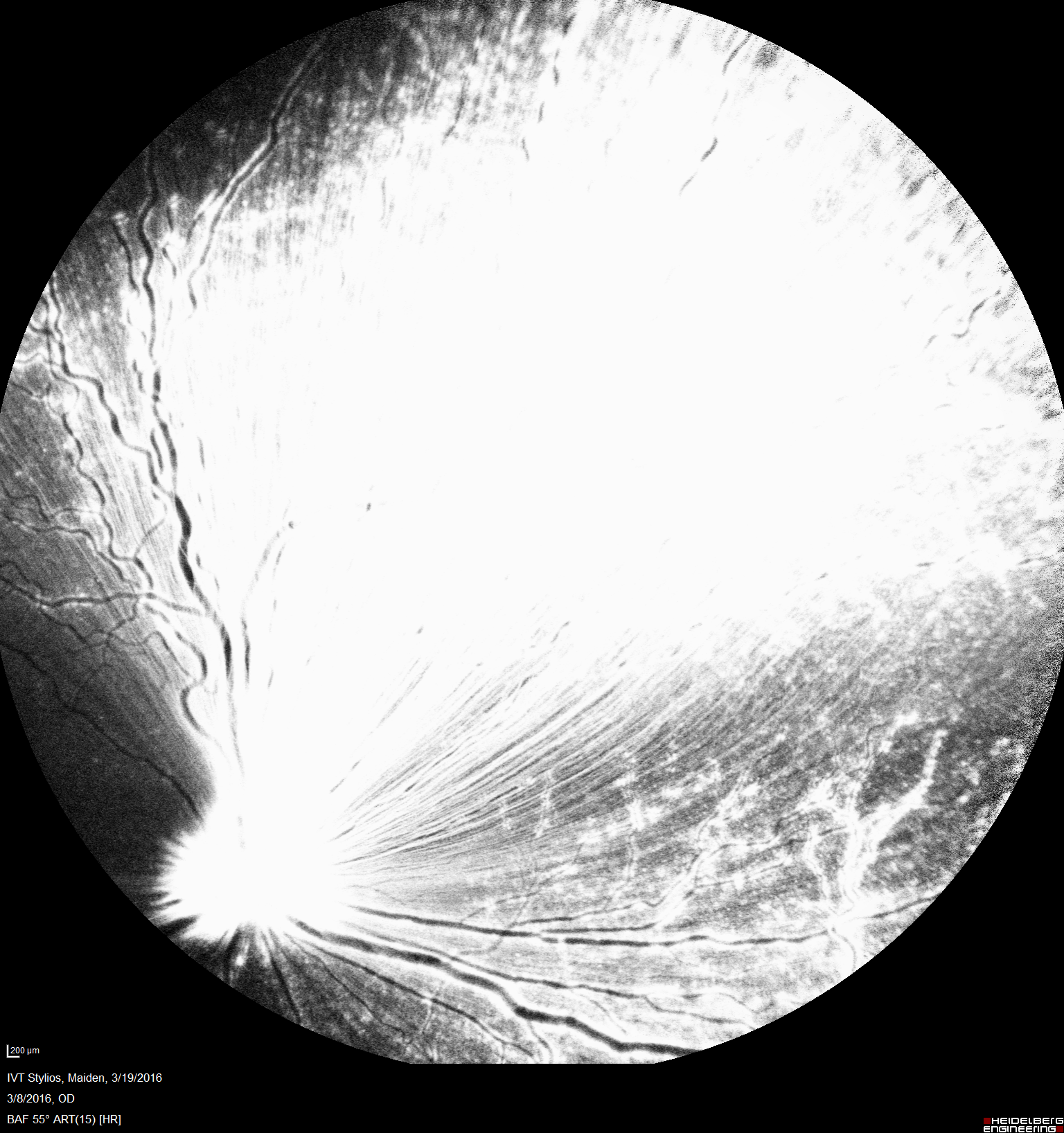

Supplement: Supplementary file 7 — Source Data for Figure 3 [file EMMM-13-e13392-s005.zip › Source_Data_File_for_Fig_3/6WPI_GL.tif]

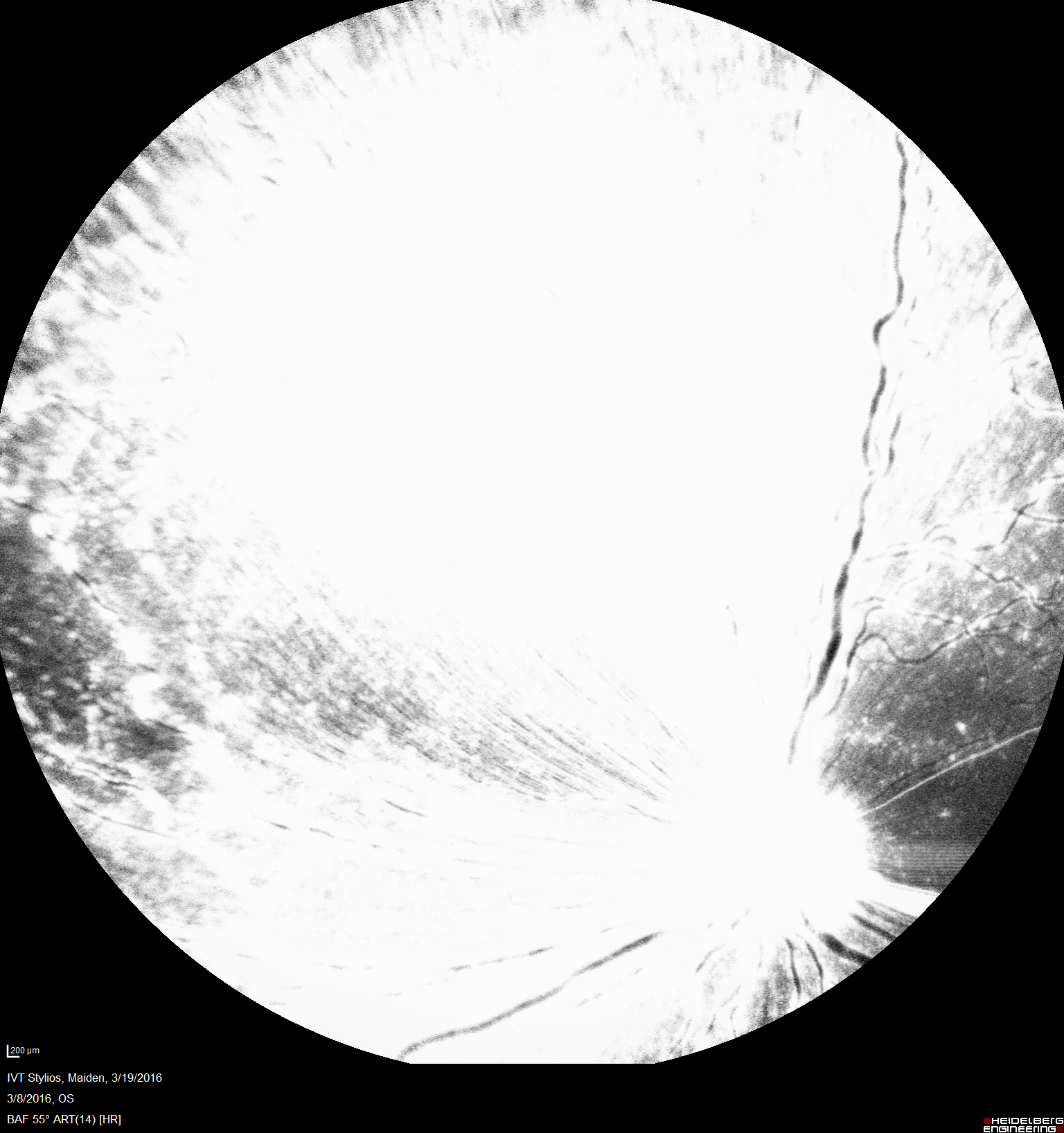

Supplement: Supplementary file 7 — Source Data for Figure 3 [file EMMM-13-e13392-s005.zip › Source_Data_File_for_Fig_3/6WPI_NN.tif]

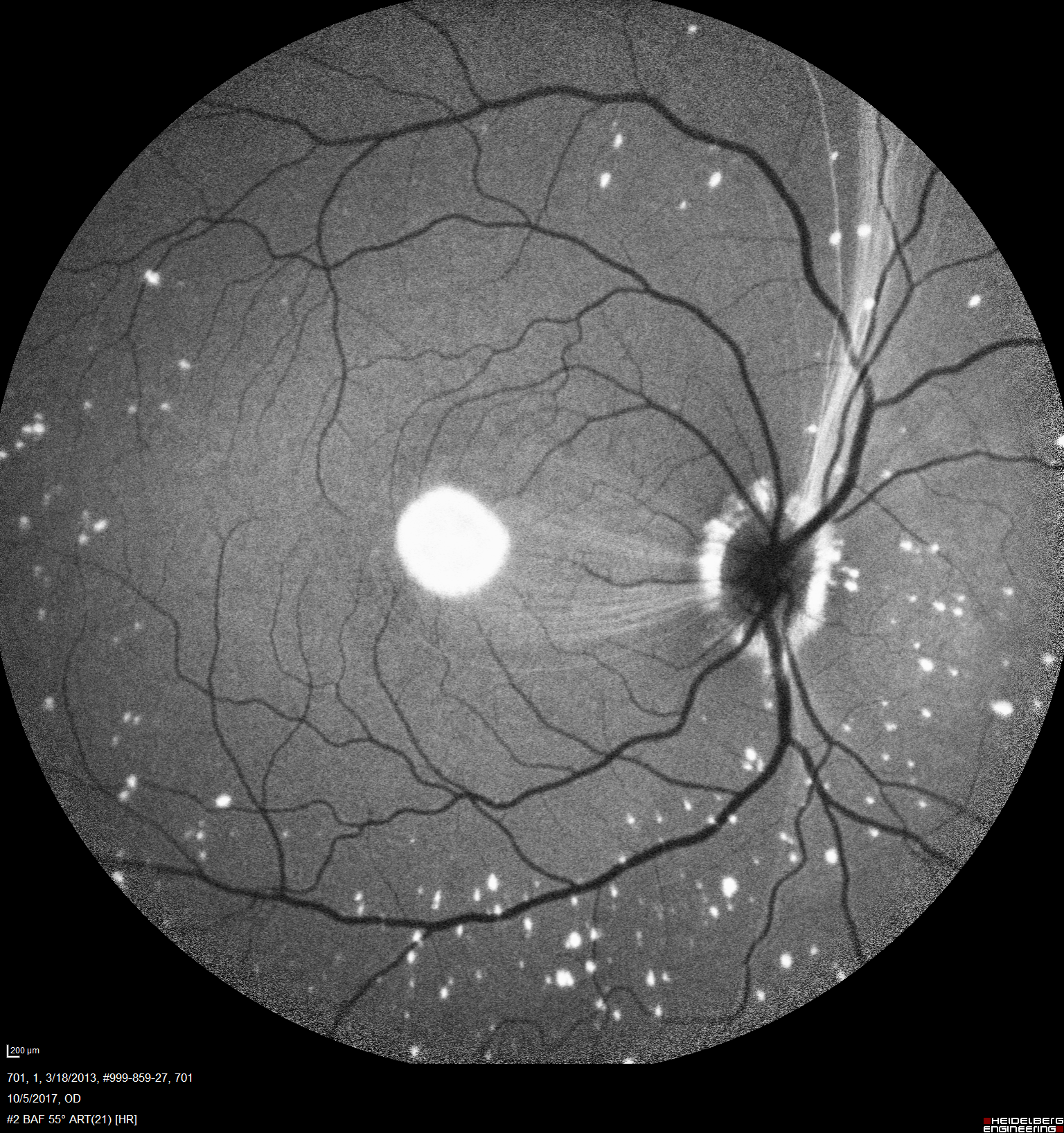

Supplement: Supplementary file 8 — Source Data for Figure 4 [file EMMM-13-e13392-s002.zip › Source_Data_File_for_Fig_4/AAV2.GL_1.tif]

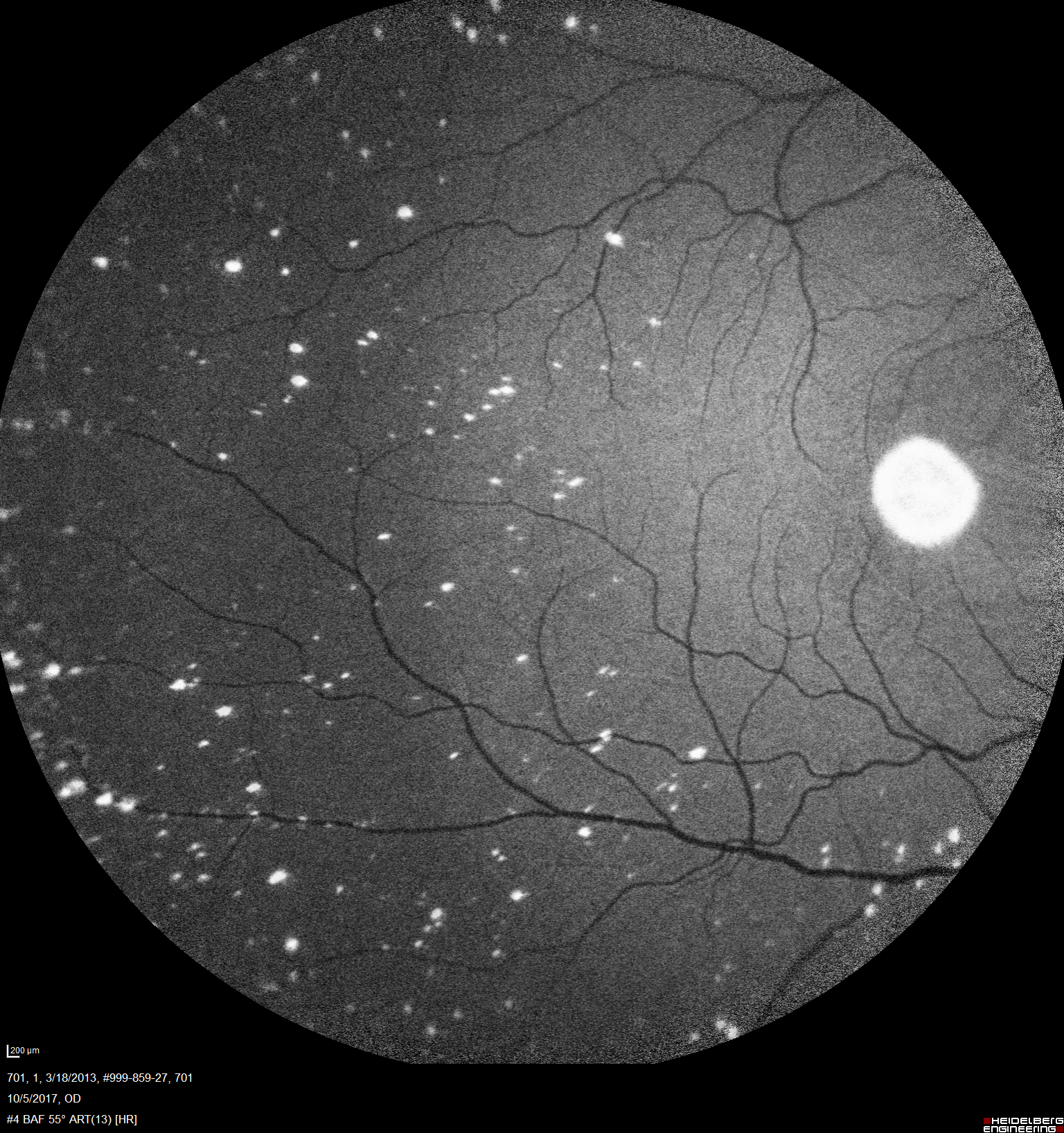

Supplement: Supplementary file 8 — Source Data for Figure 4 [file EMMM-13-e13392-s002.zip › Source_Data_File_for_Fig_4/AAV2.GL_2.tif]

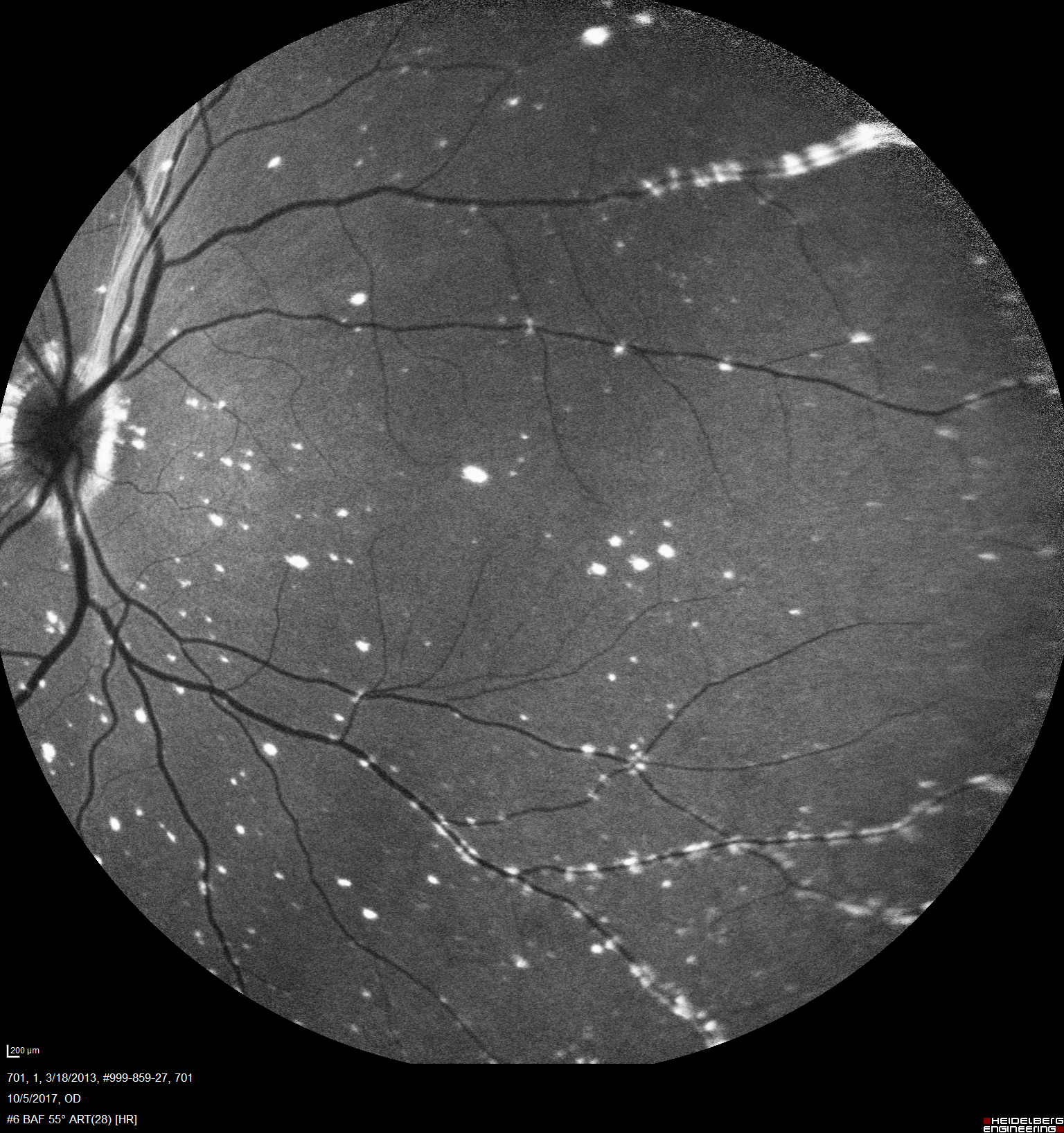

Supplement: Supplementary file 8 — Source Data for Figure 4 [file EMMM-13-e13392-s002.zip › Source_Data_File_for_Fig_4/AAV2.GL_3.tif]

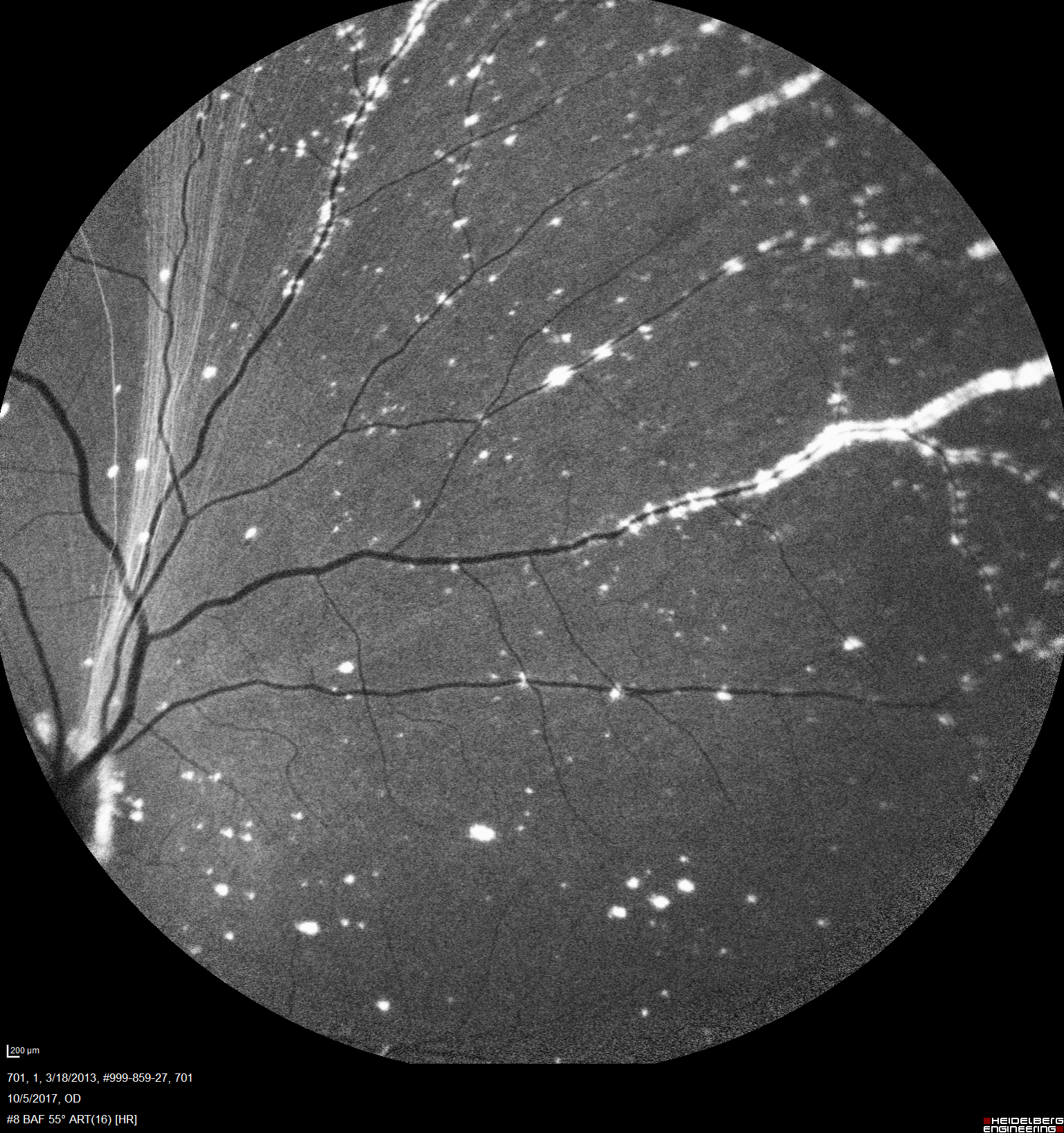

Supplement: Supplementary file 8 — Source Data for Figure 4 [file EMMM-13-e13392-s002.zip › Source_Data_File_for_Fig_4/AAV2.GL_4.tif]

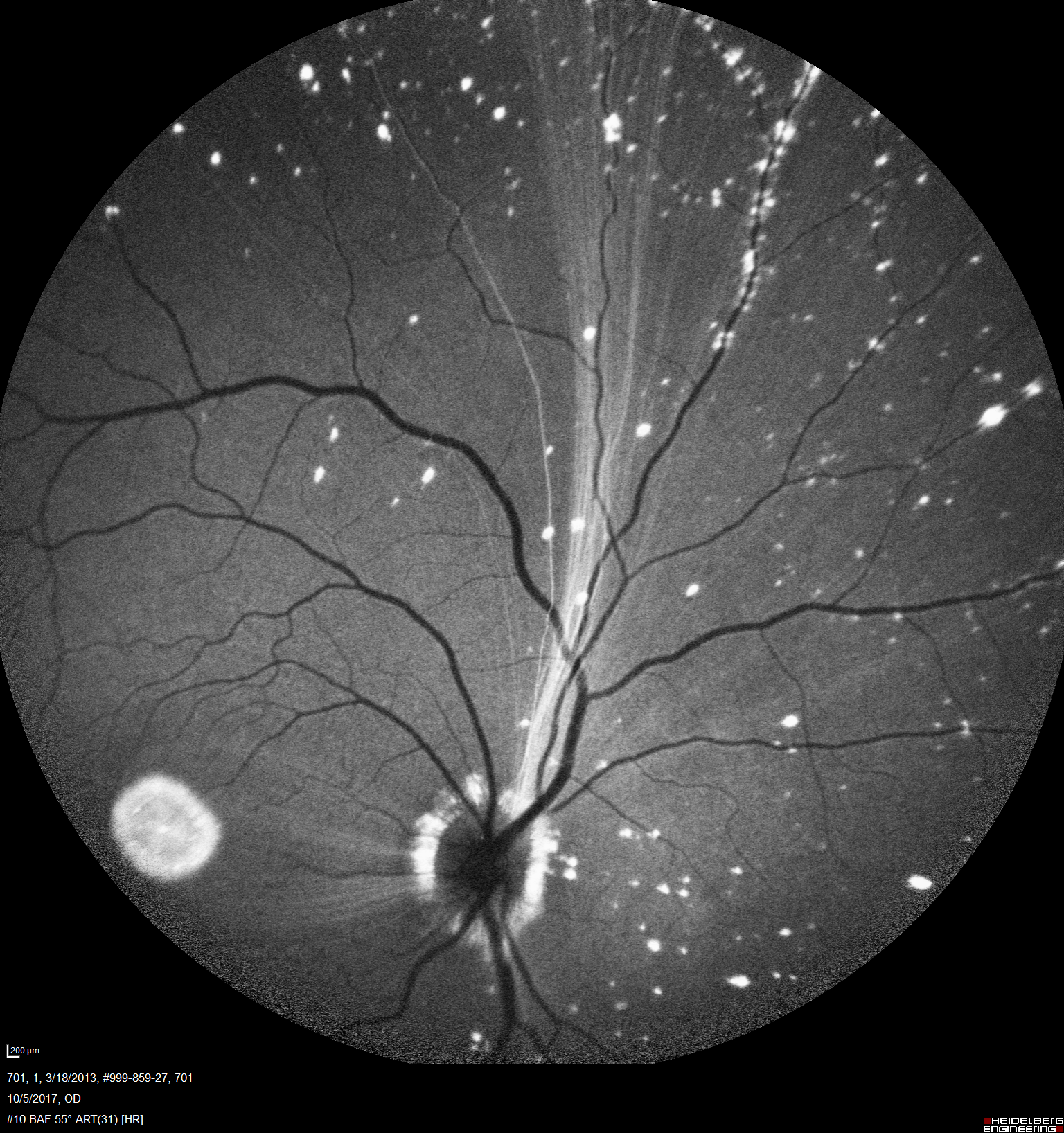

Supplement: Supplementary file 8 — Source Data for Figure 4 [file EMMM-13-e13392-s002.zip › Source_Data_File_for_Fig_4/AAV2.GL_5.tif]

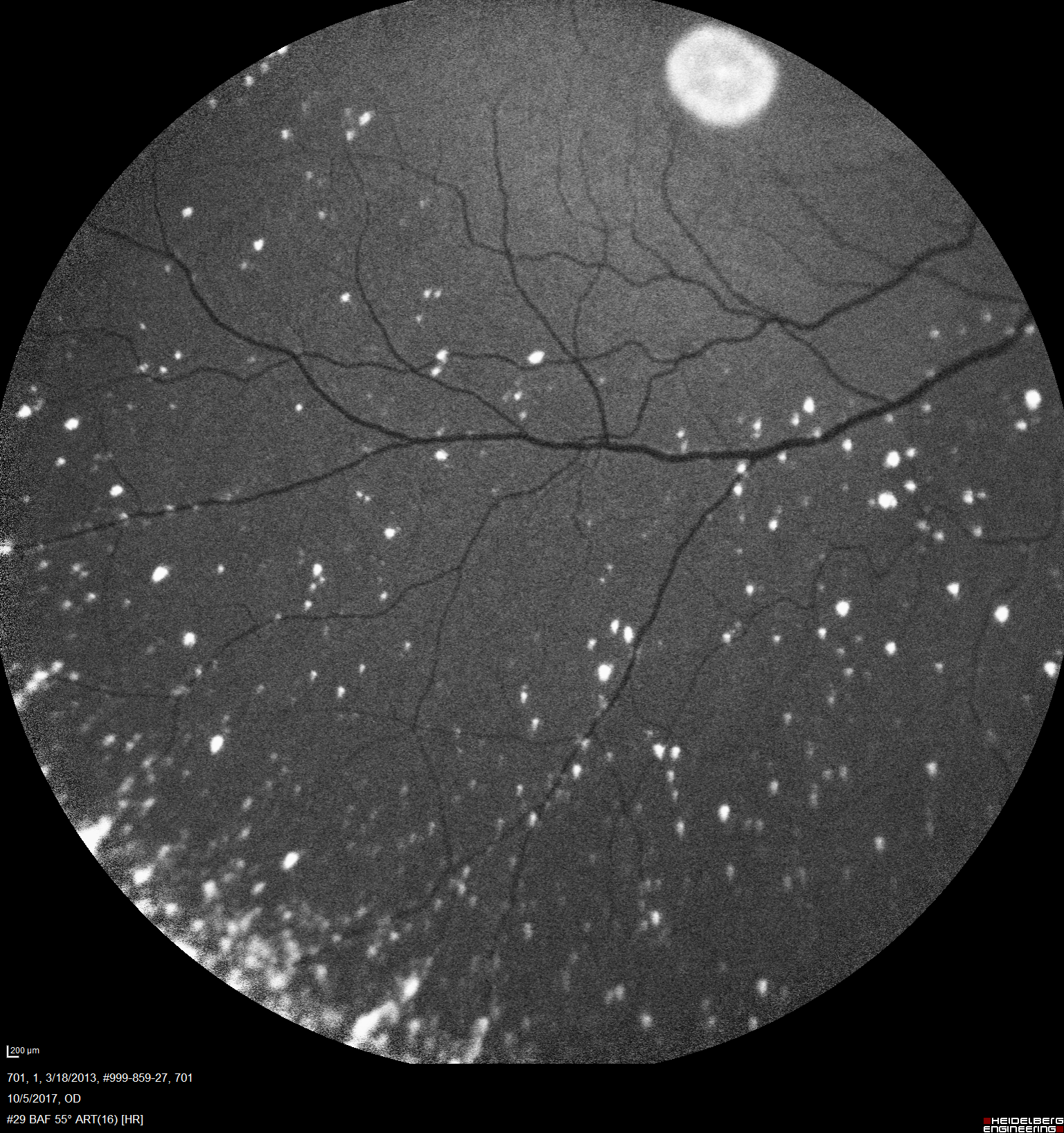

Supplement: Supplementary file 8 — Source Data for Figure 4 [file EMMM-13-e13392-s002.zip › Source_Data_File_for_Fig_4/AAV2.GL_6.tif]

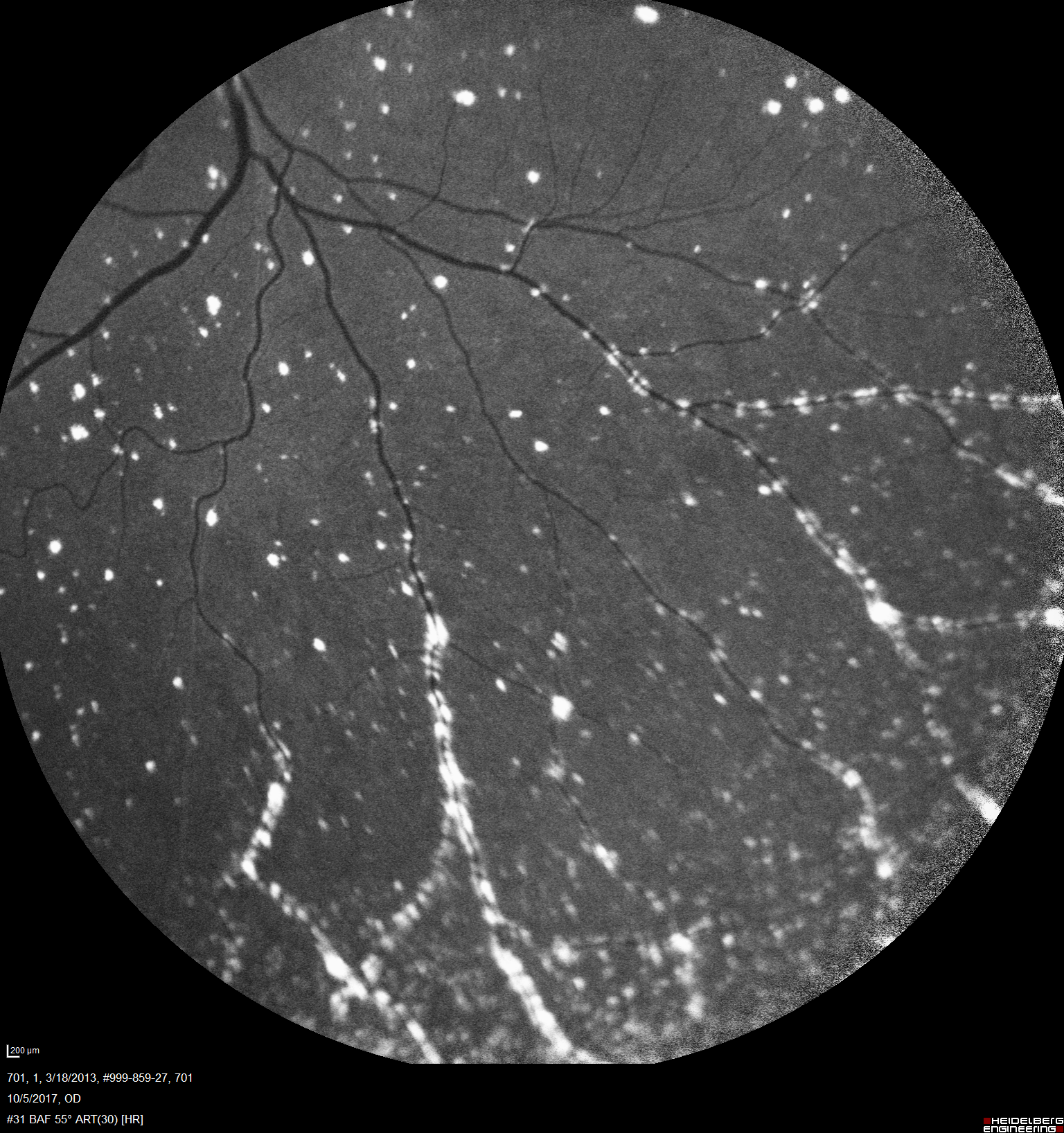

Supplement: Supplementary file 8 — Source Data for Figure 4 [file EMMM-13-e13392-s002.zip › Source_Data_File_for_Fig_4/AAV2.GL_7.tif]

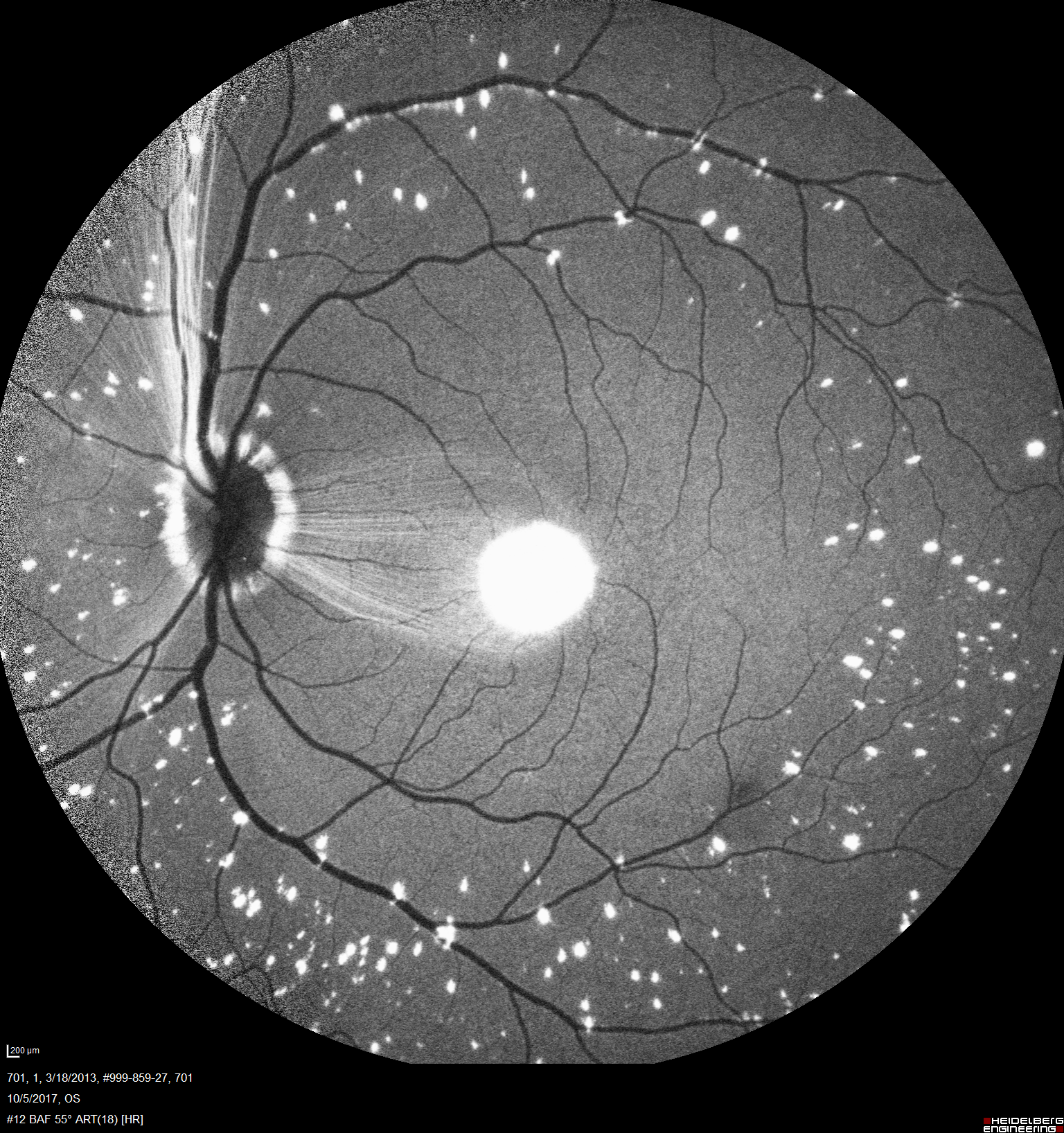

Supplement: Supplementary file 8 — Source Data for Figure 4 [file EMMM-13-e13392-s002.zip › Source_Data_File_for_Fig_4/AAV2.NN_1.tif]

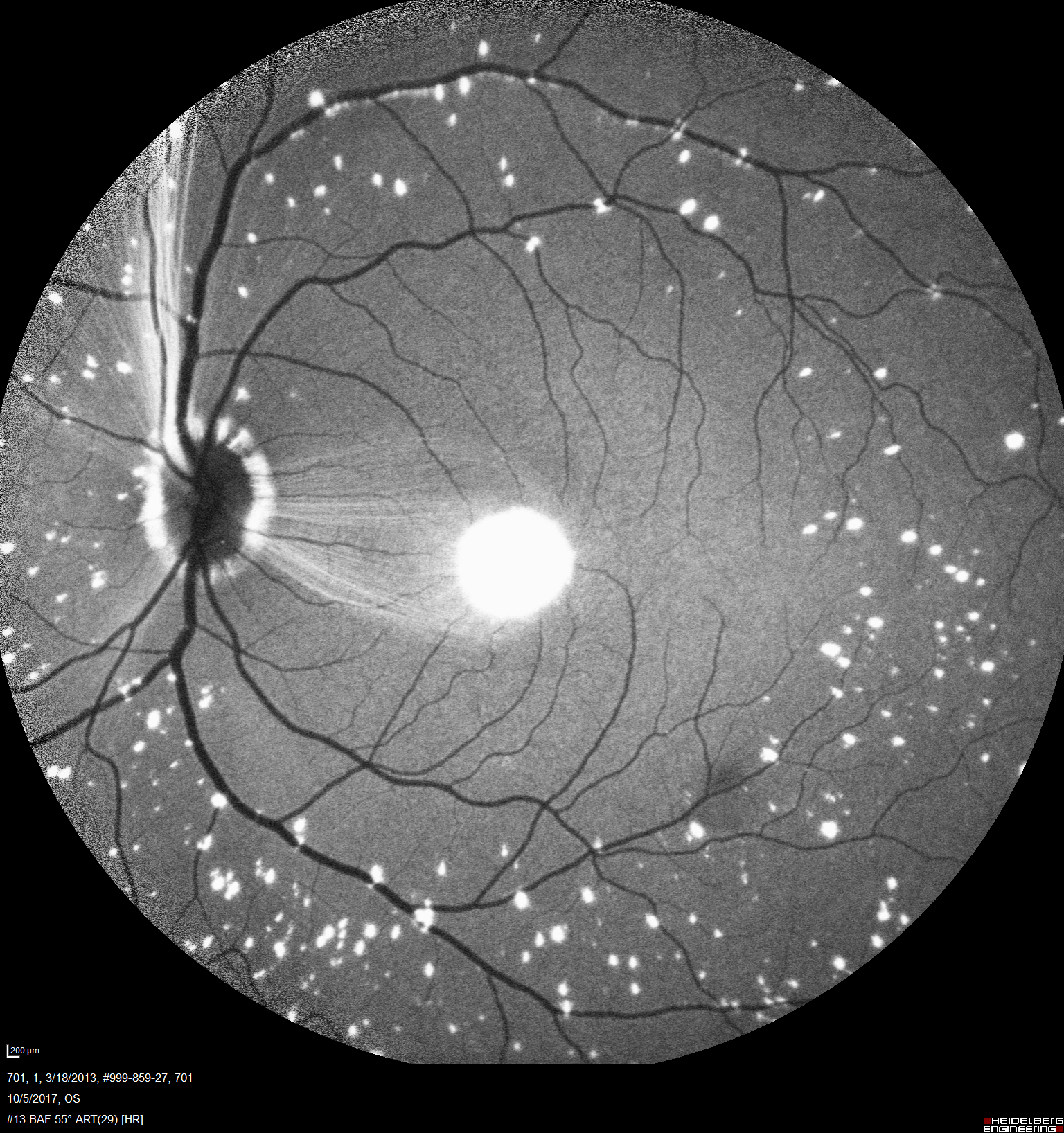

Supplement: Supplementary file 8 — Source Data for Figure 4 [file EMMM-13-e13392-s002.zip › Source_Data_File_for_Fig_4/AAV2.NN_2.tif]

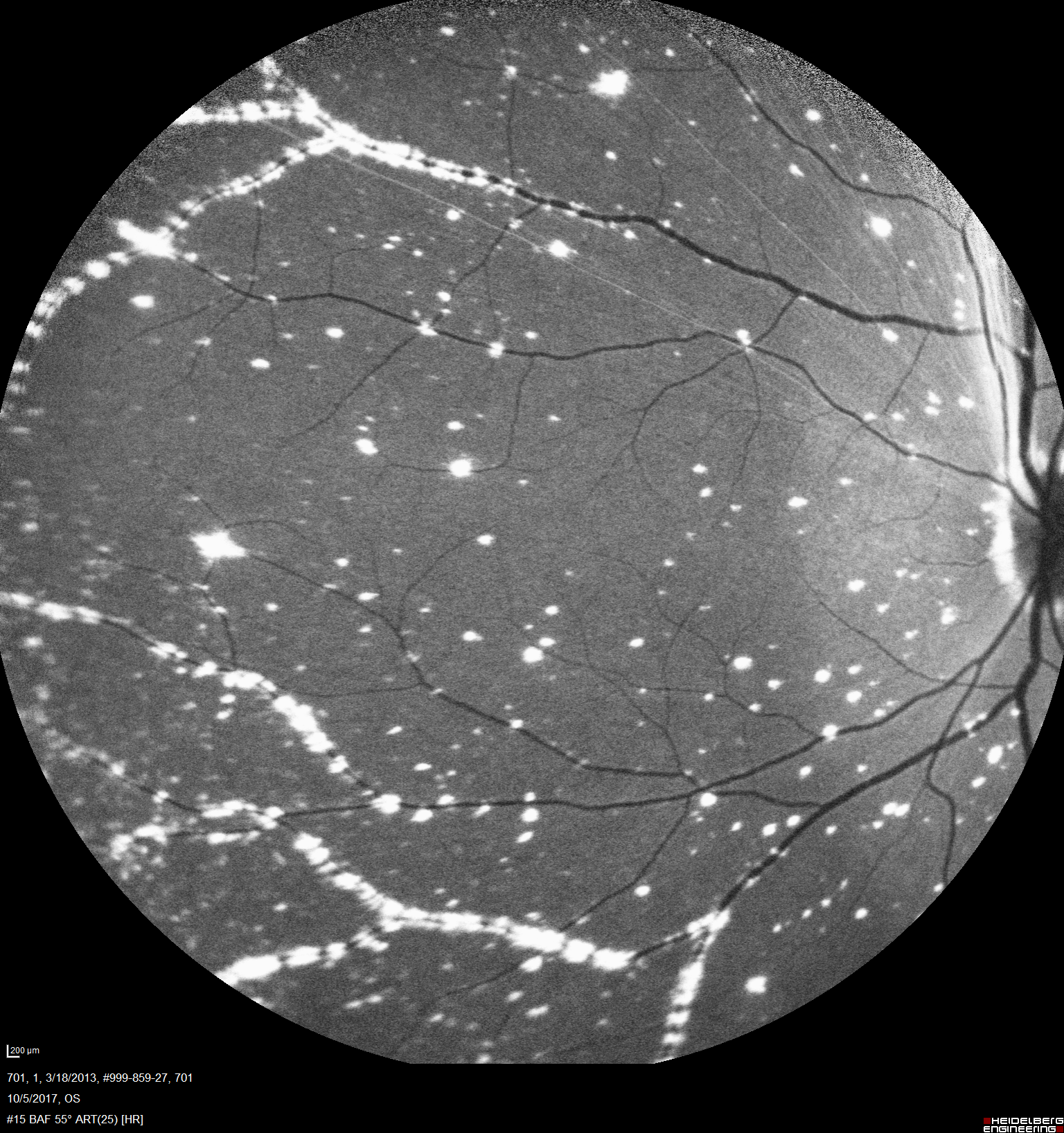

Supplement: Supplementary file 8 — Source Data for Figure 4 [file EMMM-13-e13392-s002.zip › Source_Data_File_for_Fig_4/AAV2.NN_3.tif]

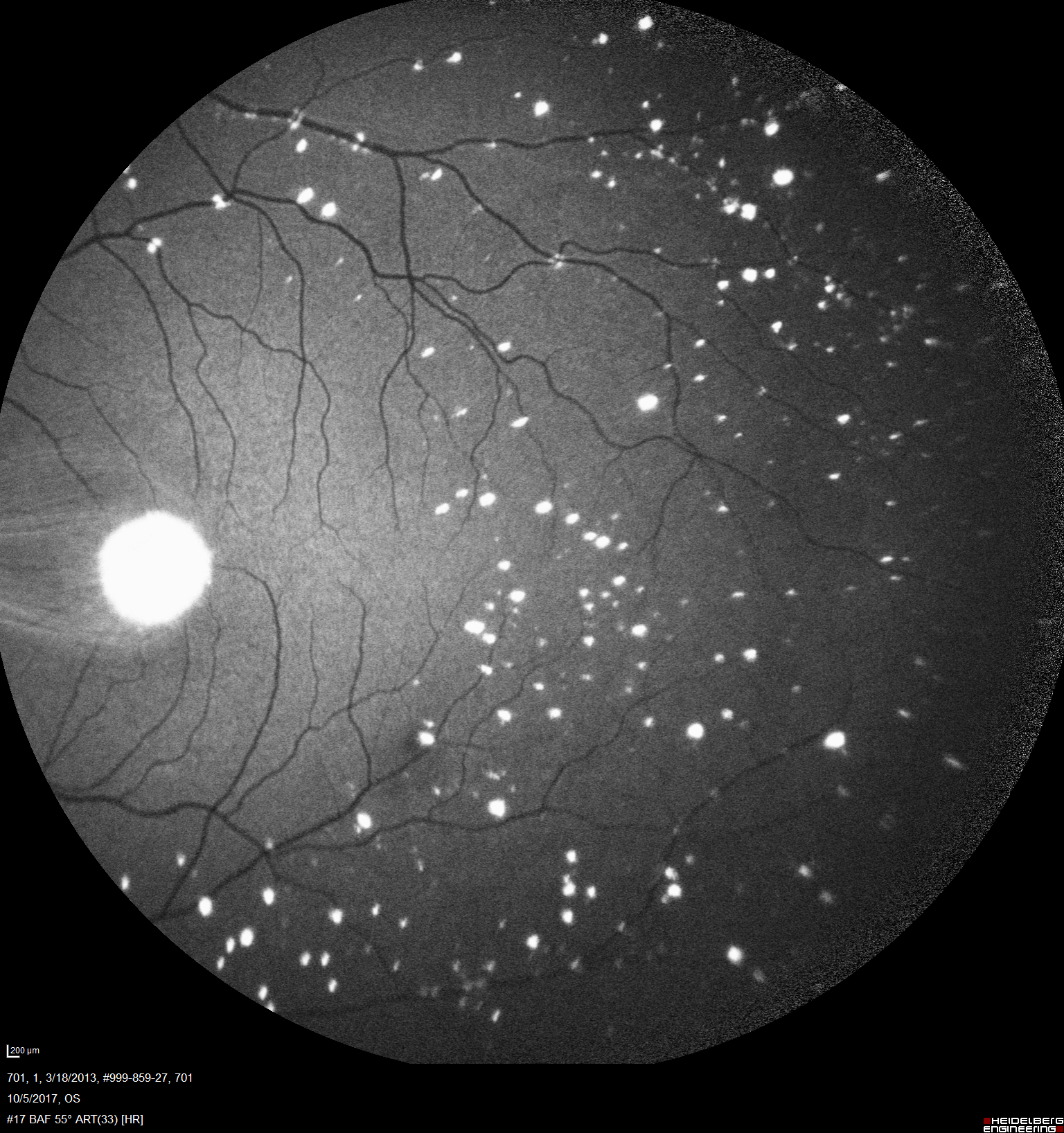

Supplement: Supplementary file 8 — Source Data for Figure 4 [file EMMM-13-e13392-s002.zip › Source_Data_File_for_Fig_4/AAV2.NN_4.tif]

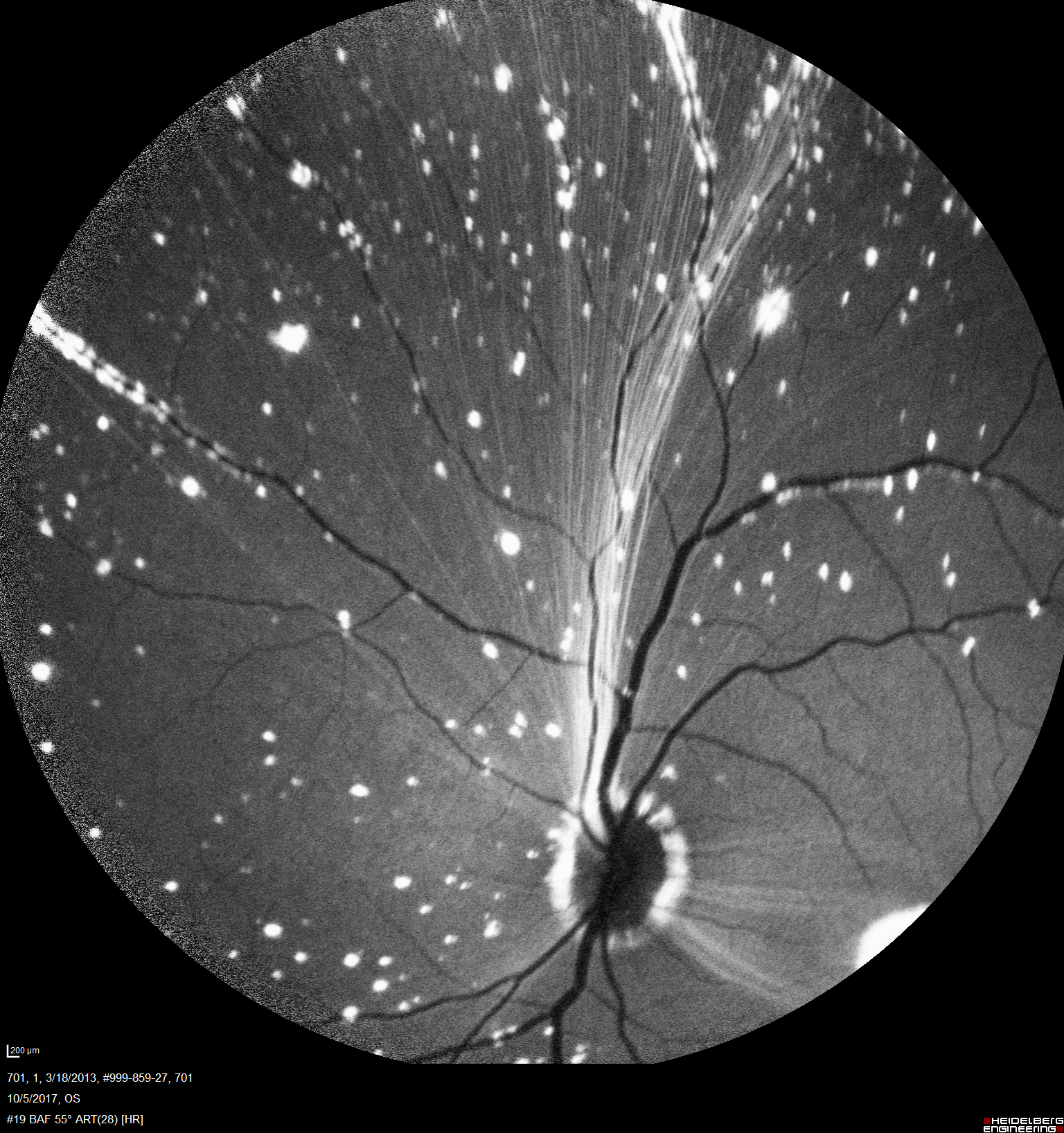

Supplement: Supplementary file 8 — Source Data for Figure 4 [file EMMM-13-e13392-s002.zip › Source_Data_File_for_Fig_4/AAV2.NN_5.tif]

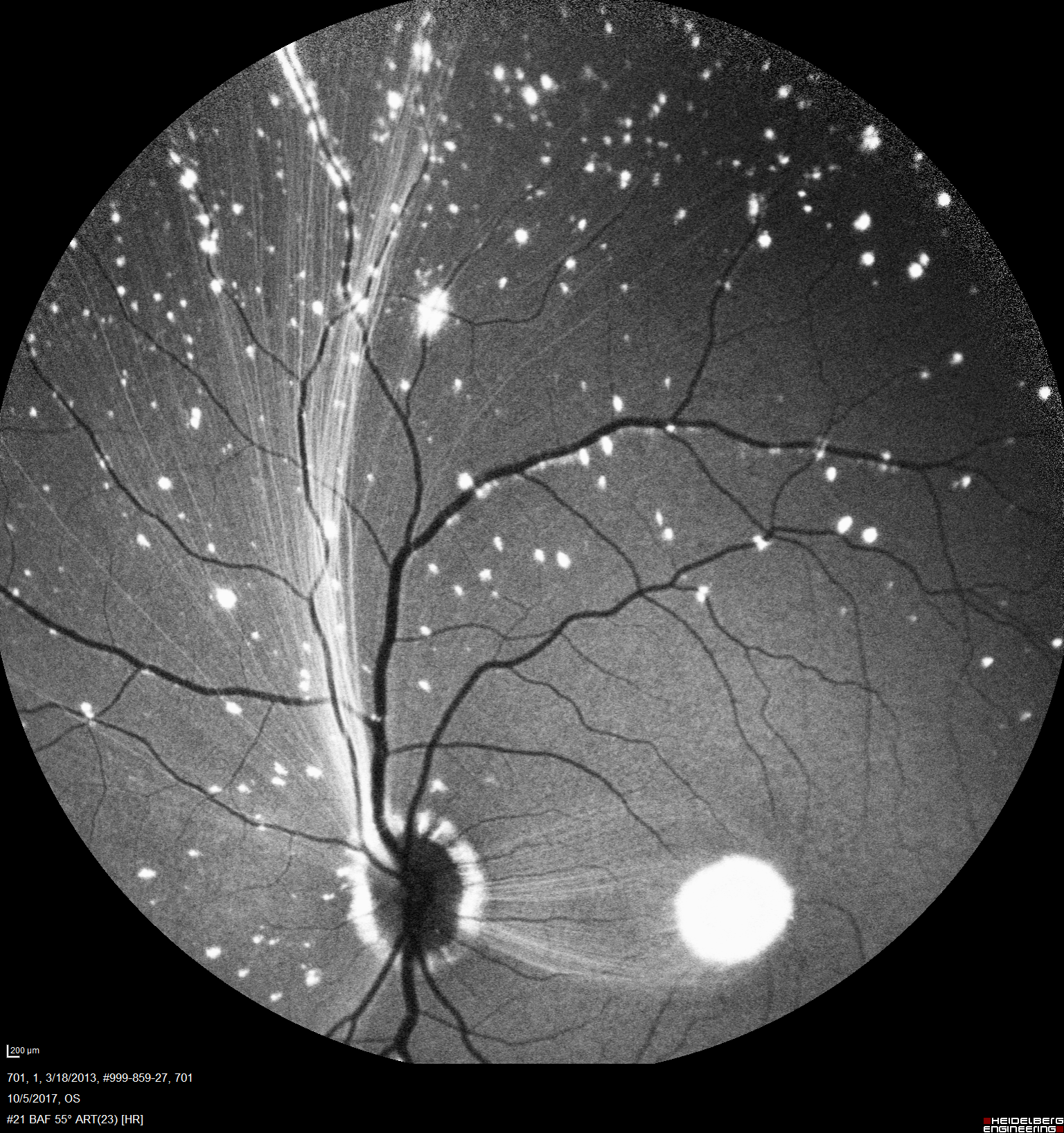

Supplement: Supplementary file 8 — Source Data for Figure 4 [file EMMM-13-e13392-s002.zip › Source_Data_File_for_Fig_4/AAV2.NN_6.tif]

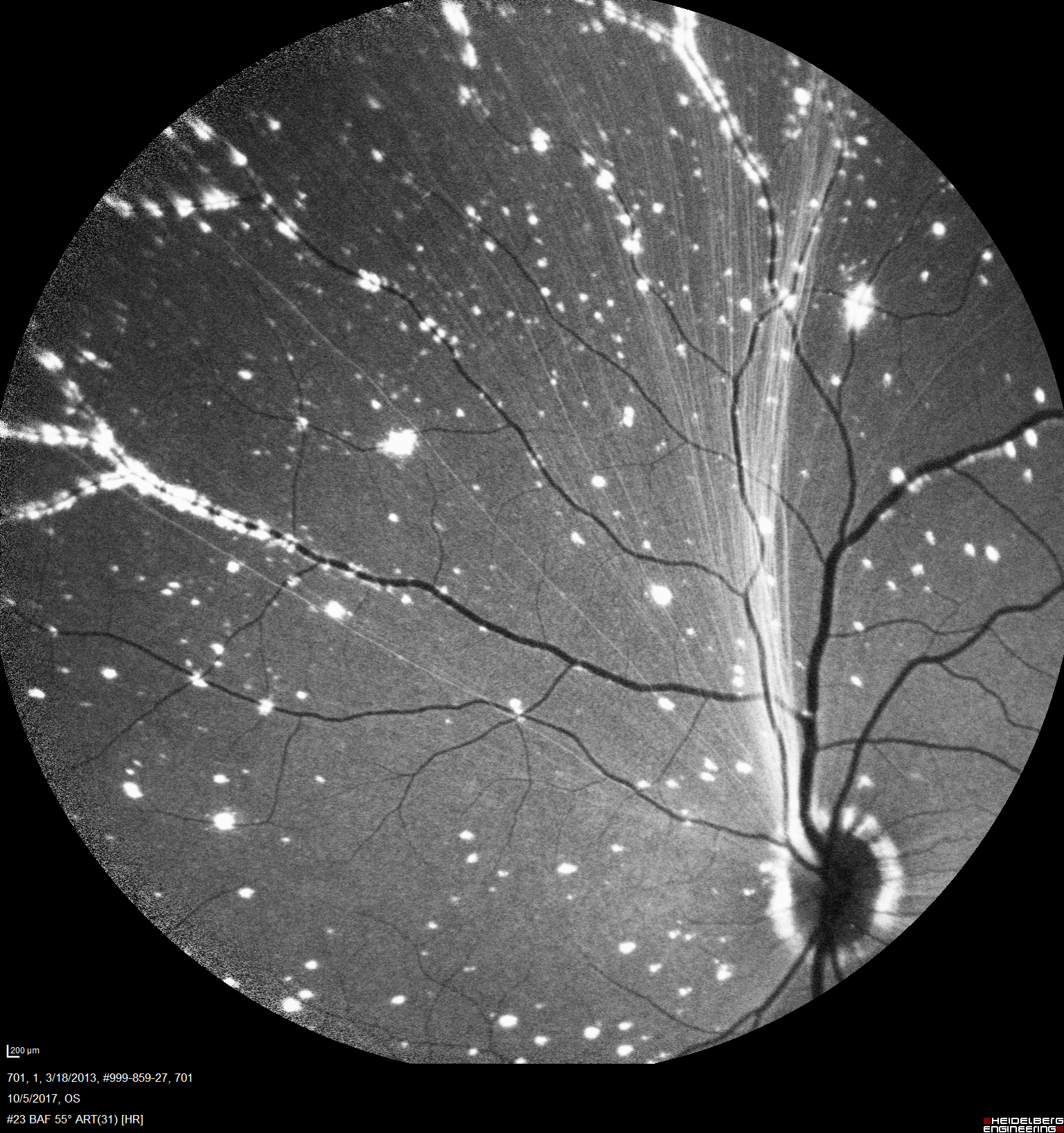

Supplement: Supplementary file 8 — Source Data for Figure 4 [file EMMM-13-e13392-s002.zip › Source_Data_File_for_Fig_4/AAV2.NN_7.tif]

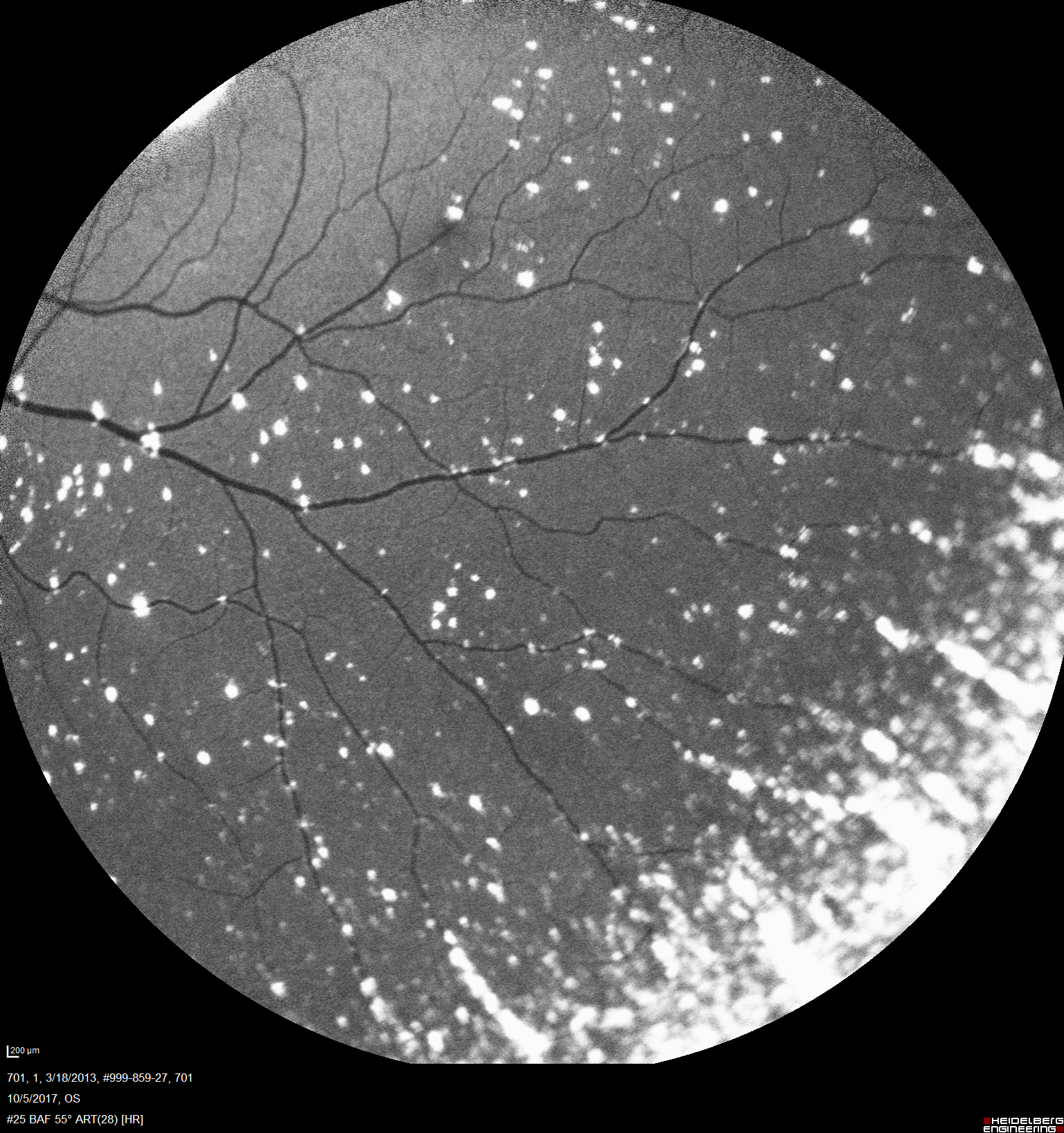

Supplement: Supplementary file 8 — Source Data for Figure 4 [file EMMM-13-e13392-s002.zip › Source_Data_File_for_Fig_4/AAV2.NN_8.tif]

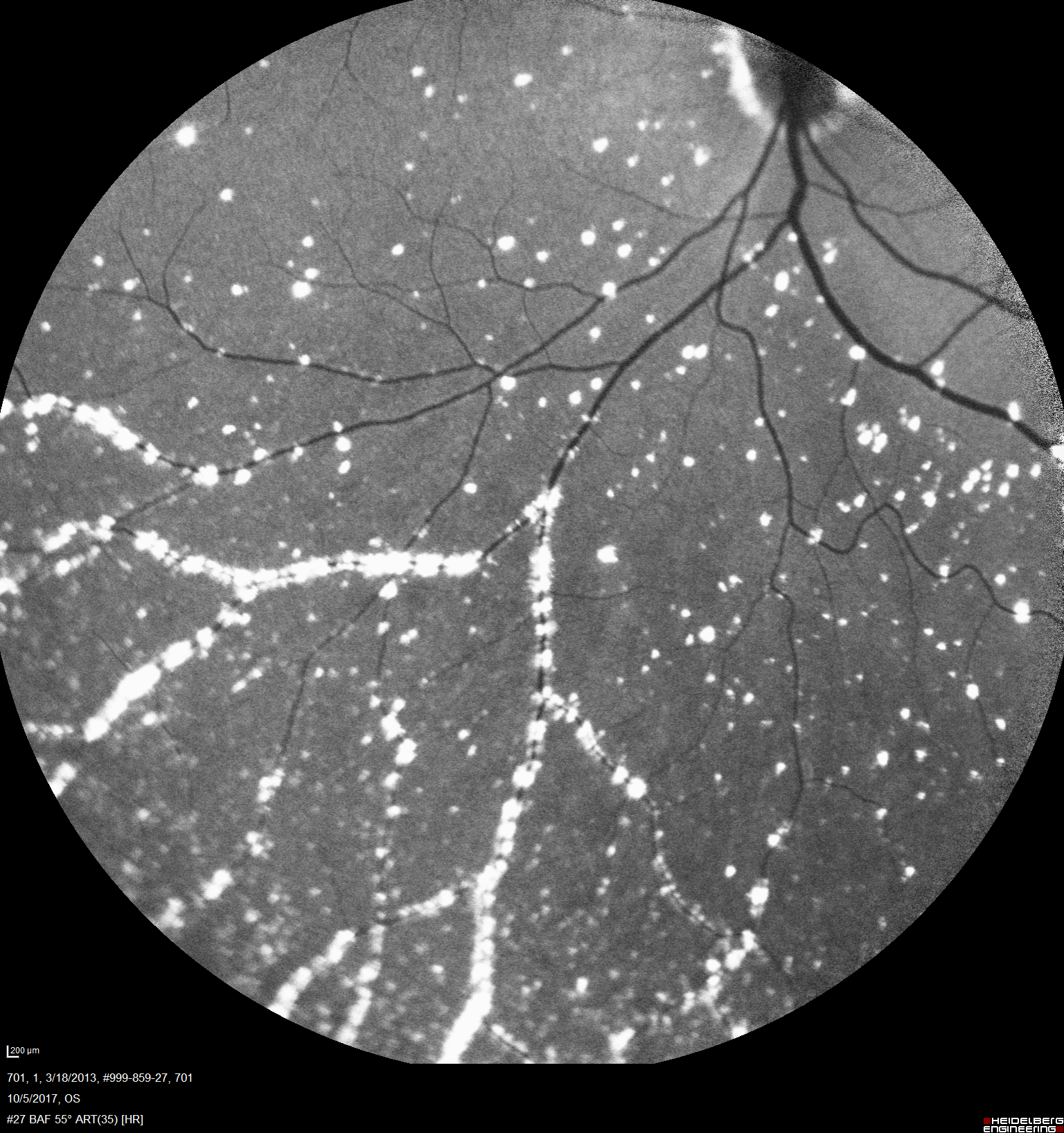

Supplement: Supplementary file 8 — Source Data for Figure 4 [file EMMM-13-e13392-s002.zip › Source_Data_File_for_Fig_4/AAV2.NN_9.tif]

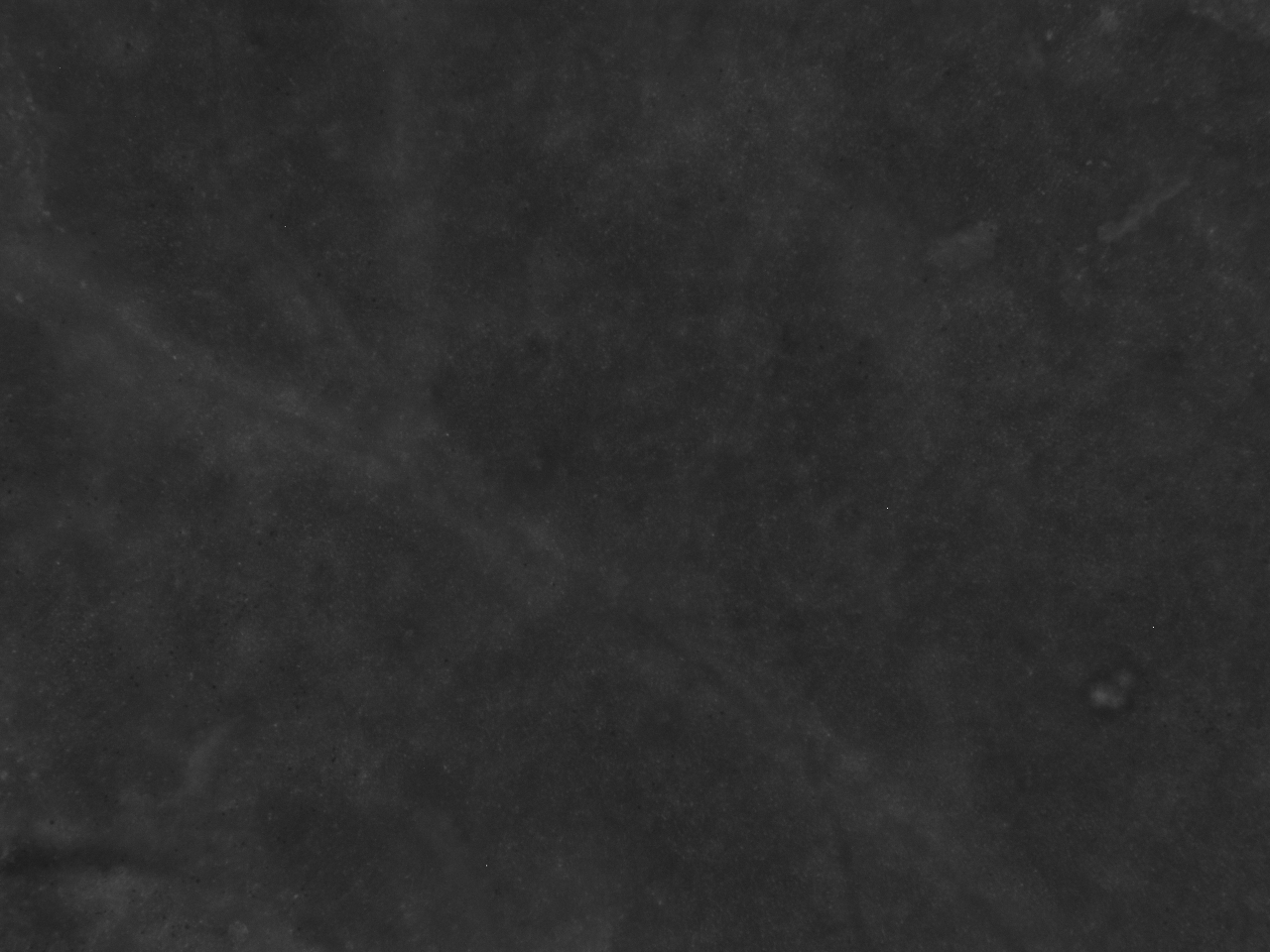

Supplement: Supplementary file 9 — Source Data for Figure 5 [file EMMM-13-e13392-s006.zip › Source_Data_File_for_Fig_5/D3_GL.tif]

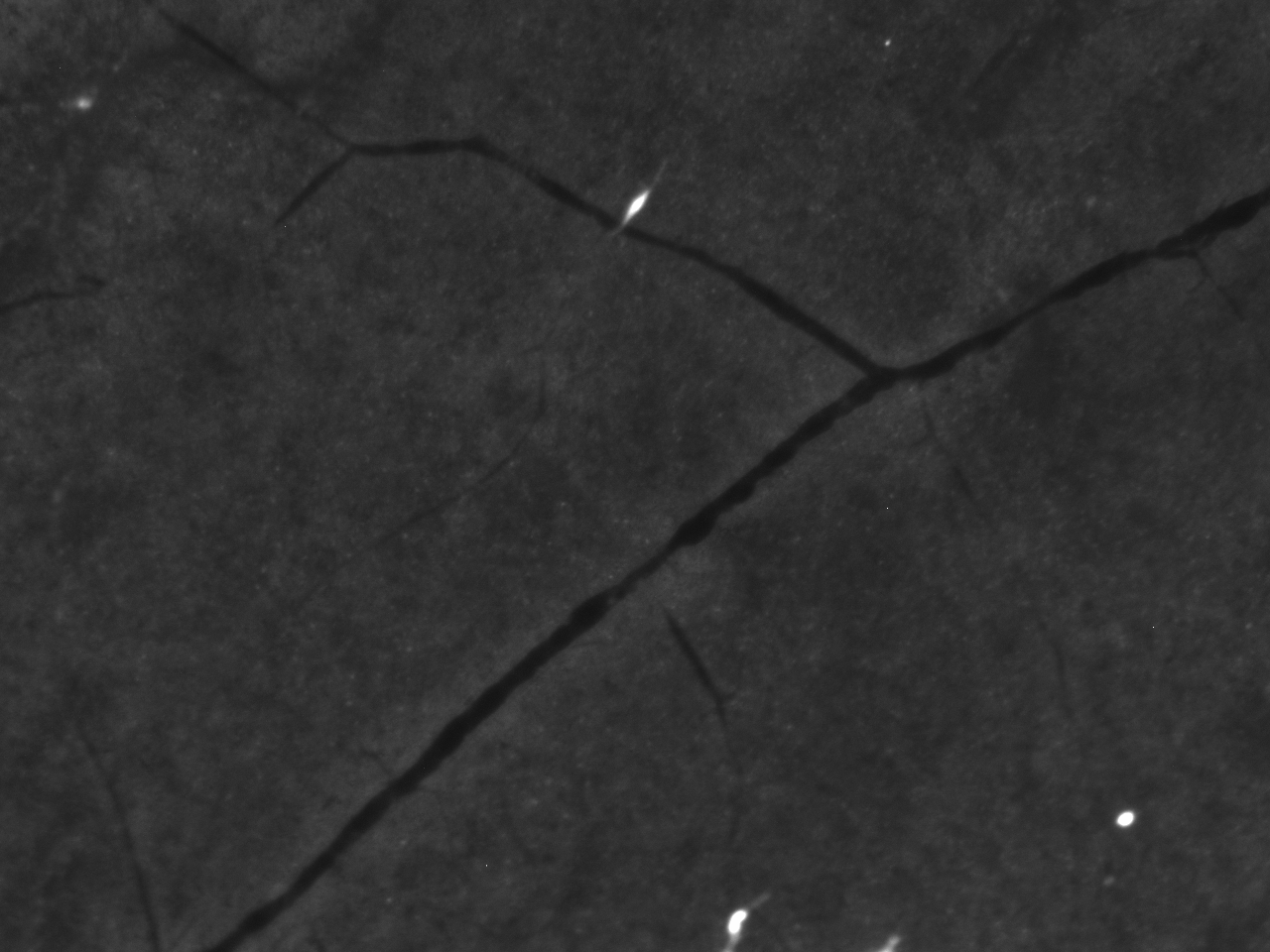

Supplement: Supplementary file 9 — Source Data for Figure 5 [file EMMM-13-e13392-s006.zip › Source_Data_File_for_Fig_5/D3_NN.tif]

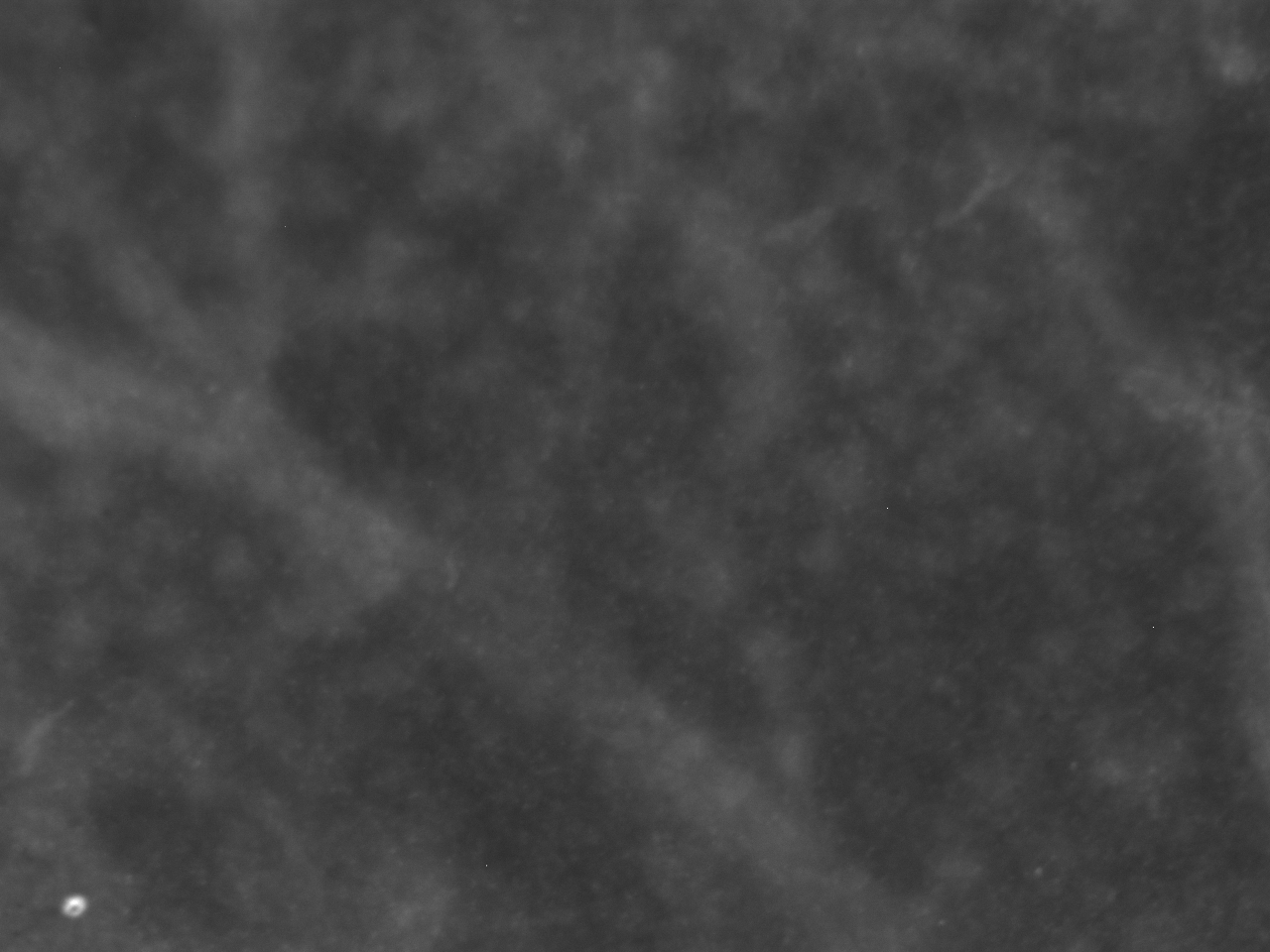

Supplement: Supplementary file 9 — Source Data for Figure 5 [file EMMM-13-e13392-s006.zip › Source_Data_File_for_Fig_5/D6_GL.tif]

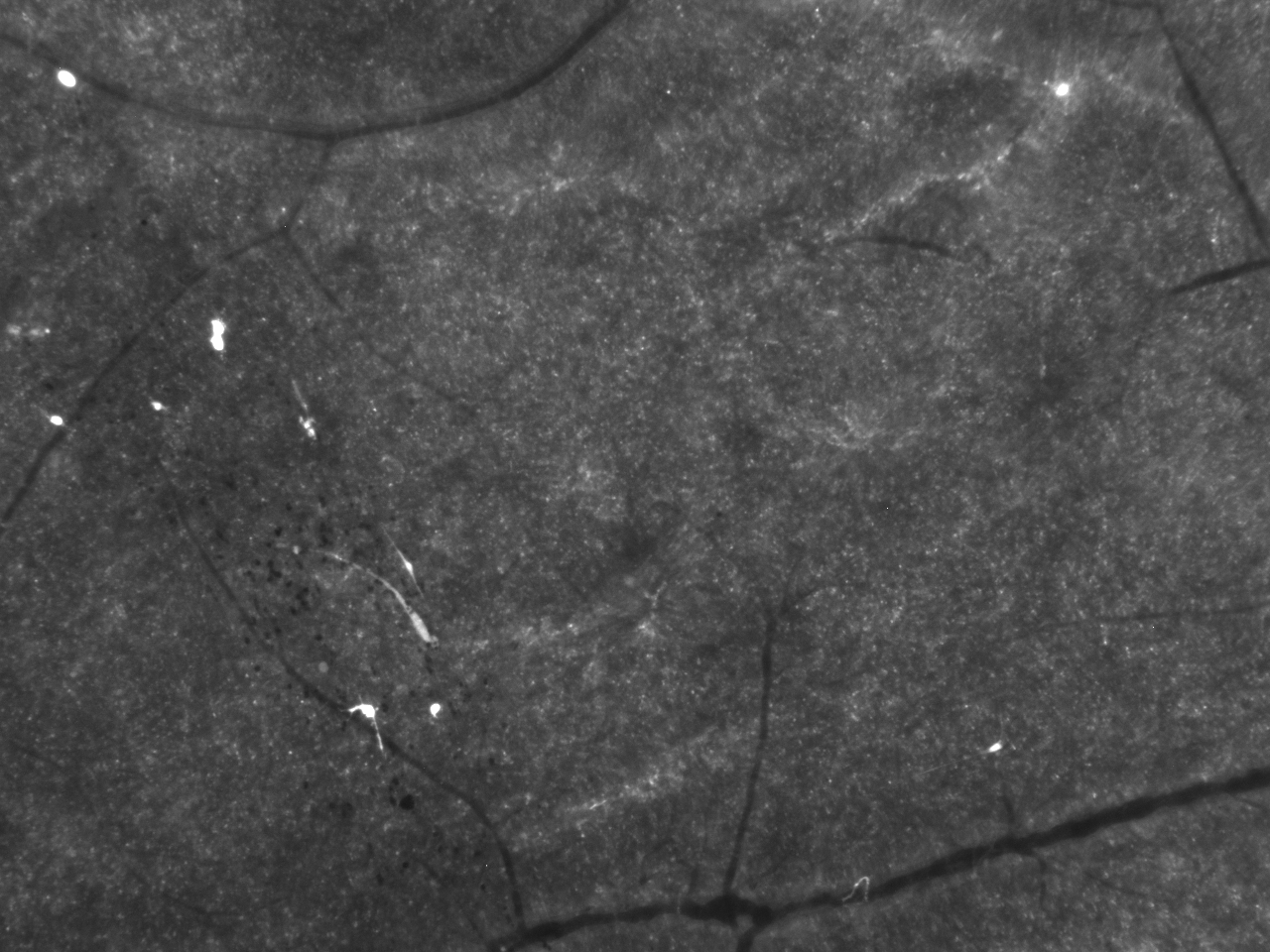

Supplement: Supplementary file 9 — Source Data for Figure 5 [file EMMM-13-e13392-s006.zip › Source_Data_File_for_Fig_5/D6_NN.tif]

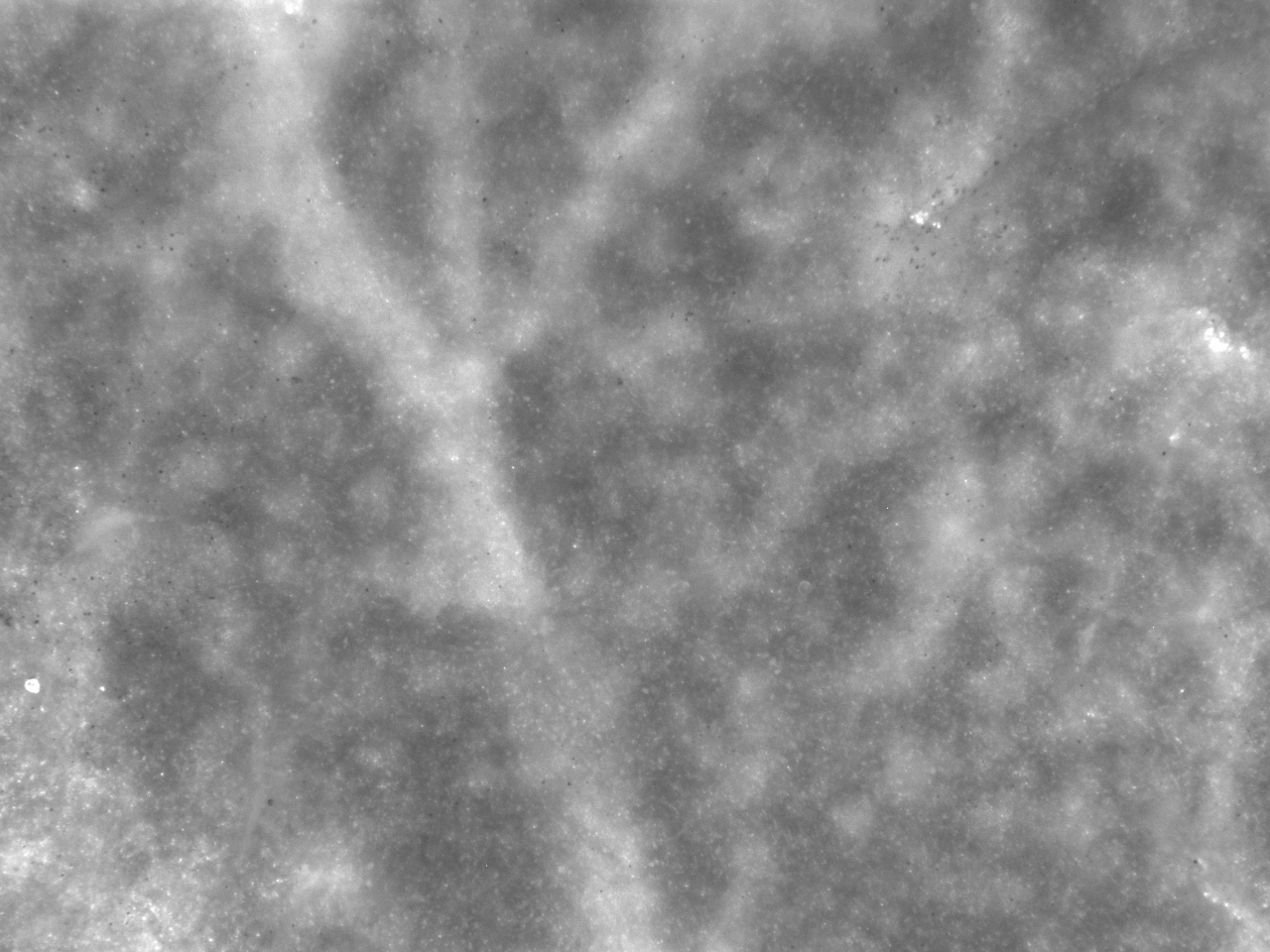

Supplement: Supplementary file 9 — Source Data for Figure 5 [file EMMM-13-e13392-s006.zip › Source_Data_File_for_Fig_5/D9_GL.tif]

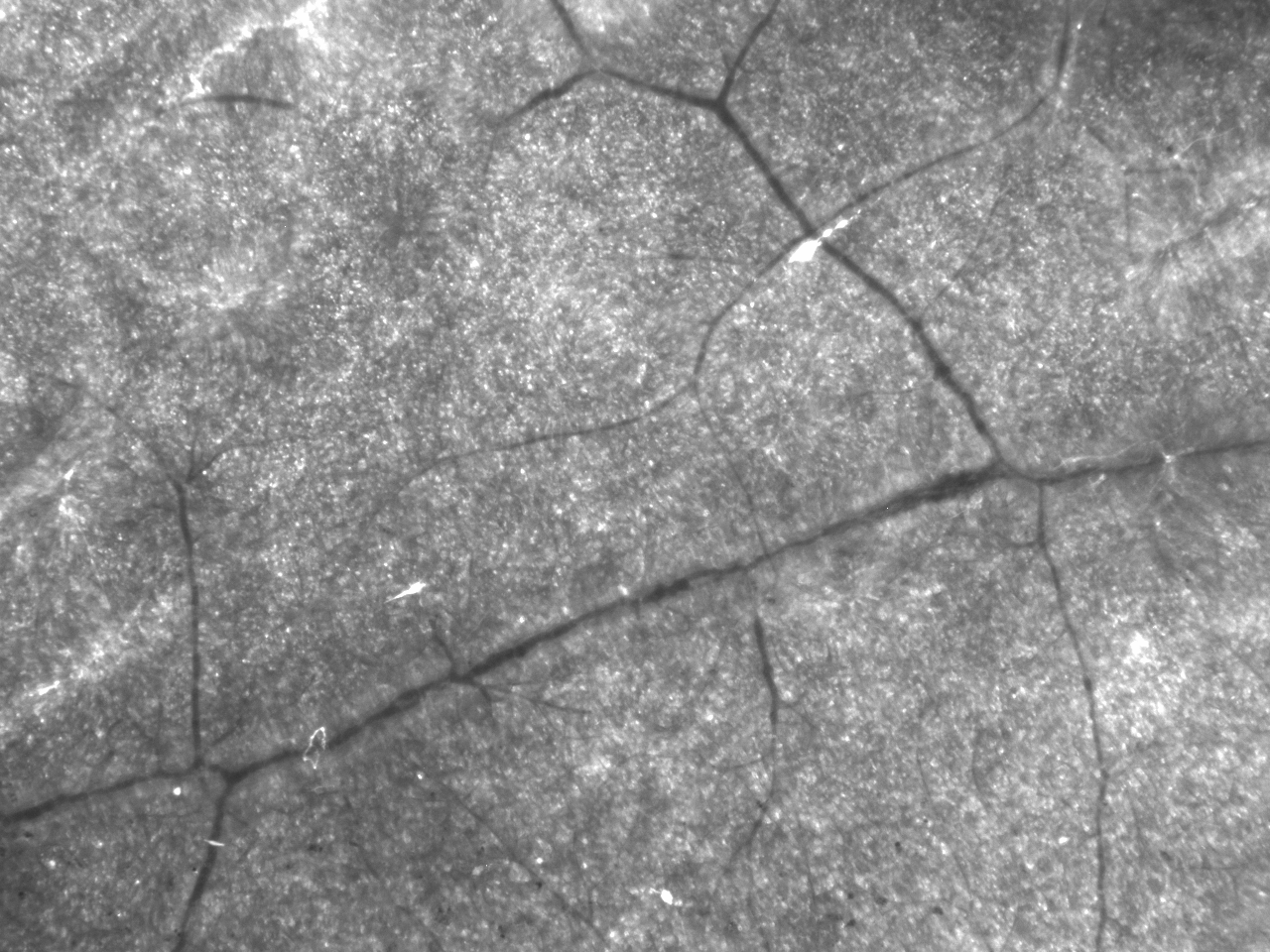

Supplement: Supplementary file 9 — Source Data for Figure 5 [file EMMM-13-e13392-s006.zip › Source_Data_File_for_Fig_5/D9_NN.tif]
